# Strain Effects on the Adsorption of Water on Cerium Dioxide Surfaces and Nanoparticles: A Modeling Outlook

**DOI:** 10.1021/acs.jpcc.4c04172

**Published:** 2024-10-17

**Authors:** Sidra Munir, Khoa Minh Ta, Thomas Smith, Lisa J. Gillie, David J. Cooke, Stephen C. Parker, Marco Molinari

**Affiliations:** † Department of Physical and Life Sciences, University of Huddersfield, Queensgate, Huddersfield HD1 3DH, U.K.; ‡ Department of Chemistry, University of Bath, Claverton Down, Bath BA2 7AY, U.K.

## Abstract

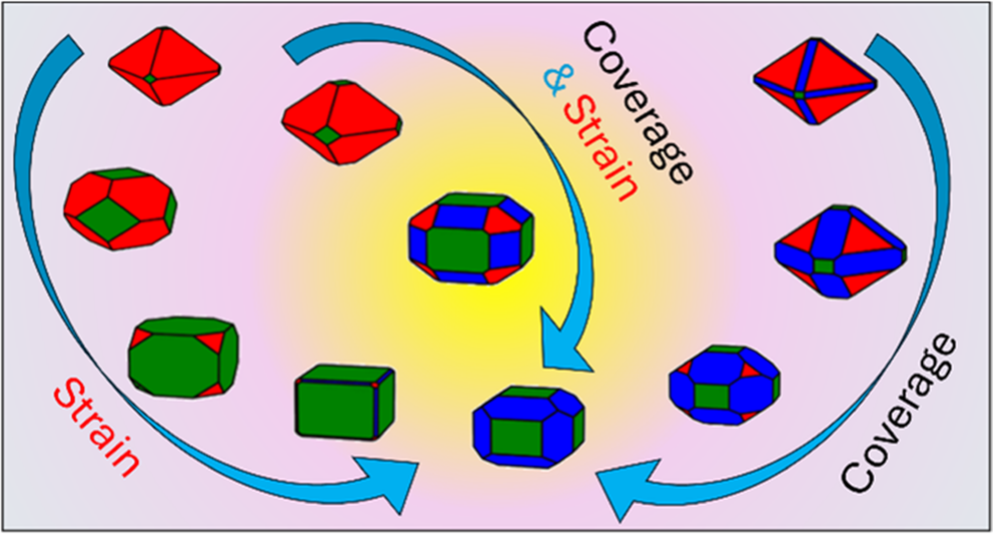

Nanocrystalline ceria exhibits significant redox activity and oxygen storage capacity. Any factor affecting its morphology can tune such activities. Strain is a promising method for controlling particle morphology, whether as core@shell structures, supported nanoparticles, or nanograins in nanocrystalline ceria. A key challenge is to define routes of controlling strain to enhance the expression of more active morphologies and to maintain their shape during the lifespan of the particle. Here, we demonstrate a computational route to gain insights into the strain effects on particle morphology. We use density functional theory to predict surface composition and particle morphology of strained ceria surfaces, as a function of environmental conditions of temperature and partial pressure of water. We find that adsorbed molecular water is not sufficient to shift stability and as such under all compressive and tensile strains studied, the most stable particle is of octahedral shape, similarly to the unstrained case. When dissociative water is involved at the surfaces of the particle, then the most stable particle morphology changes under high water coverage and tensile strain to cuboidal or truncated cuboidal shapes. This shift in shape is due to strain effects that affect the strength of water adsorption.

## Introduction

Cerium dioxide (CeO_2_) is catalytically active and has a high ionic conductivity, which makes it an ideal and effective material in catalysis and energy applications. As a catalyst, CeO_2_ has found applications in reforming, three-way catalysts, and oxidation of volatile organic compounds, while as a solid electrolyte, it has found applications in solid oxide fuel cells.^1,2^ Biomedical applications are the latest development and show the flexibility of CeO_2_, as it displays enzyme mimetic activities such as superoxide dismutase and catalase.^3,4^

CeO_2_ is an oxygen buffer (i.e., generation of oxygen vacancies) with redox activity (i.e., Ce^3+^/Ce^4+^). Any factor that can tweak such equilibria has the potential to inherently change the materials properties. Strain is an efficient method to attain remarkable performance in nanomaterials.^5^ In particular, lattice strain is an effective method for increasing oxygen storage capacity in ultrathin CeO_2−δ_, ionic conductivity in Gd_0.1_Ce_0.9_O_2−δ_/Er_2_O_3_^6^ and 8YSZ/CeO_2_^7^ films, and in La/Gd/Yb substituted CeO_2_,^8^ electronic transport in Pt/BaTiO_3_/CeO_2_ films,^9^ enhancing catalytic properties of PdCu/CeO_2_^10^ and CeO_2_/Mn_3_O_4_^11^ nanocrystals, and nanozymatic activity of ultrathin CeO_2_ antioxidants.^12^

Lattice strain is inevitable in supported films and in core@shell structures. It occurs at an interface due to the lattice mismatch of two different materials (heterogeneous interface), or of two surfaces of the same material (homogeneous interface). Coherent interfaces form when strain can be accommodated, and the two materials remain single crystals. There is however a critical point beyond which lattice strain is released via the formation of lattice defects, defect clustering, dislocations and interfaces or grain boundaries, forming incoherent interfaces.^13^

Most of the information on strained CeO_2_ is related to supported films as they are synthesized with greater experimental control. However, contrasting findings do not provide an unambiguous relationship between lattice strain and a material’s properties, hindering the design of effective strained catalysts.

In single-crystalline strained nanofilms of ∼3 nm thickness, CeO_2_(100)/STO(001) (2.1% strain) and CeO_2_(100)/YSZ(001) (−5.6% strain), both tensile and compressive biaxial strain induce a tetragonal distortion, with a 4-fold increase in the concentration of Ce^3+^ following a decrease in the energy of formation of oxygen vacancies (Vo).^14^ Although compressive strain does not favor clustering of Vo in CeO_2_(111)/Pt(111) (−1.3% strain),^15^ self-assembly of Vo is seen in CeO_2_/Rh(111) films (−1.5% strain) under reducing atmospheres, most likely from the strain-induced reduction in the formation energy of Vo.^16^

Density functional theory (DFT) calculations involving strained CeO_2_ are mainly focused on the formation of Vo. Compressive strain destabilizes and tensile strain stabilizes Vo formation,^17^ as the latter can easily accommodate Vo.^18^ The electronic density of states show that compressive/tensile strain results in an upshift/downshift of the Ce 4f-band, which underlies the destabilization/stabilization of Vo formation.^19^ Tensile strain induces a narrowing of the band gap of both bulk CeO_2_^20^ and the {111} stoichiometric surface,^21^ while in the latter, compressive strain has the opposite effect. The narrowing of the band gap is also seen in reduced unstrained CeO_2_ bulk due to the formation of Ce^3+^ and Vo, which induce lattice expansion.^22^

Compressive strain increases the water splitting reaction (WSR) activity of CeO_2_ as well as reducing the free energy of formation of surface hydroxyl groups.^23^ Indeed, oxygen vacancies and hydroxyl groups are stabilized by tensile strain but destabilized by compressive strain. Again, this is due to a downshift of the Ce 4f orbital energy under tensile strain, which can accommodate the larger size of Ce^3+^ ions compared to Ce^4+^ ions.^23^ Tensile strain increases the reactivity of the CeO_2_(111) surface, by either lowering the diffusion barrier of oxygen vacancies or increasing the reactivity toward water splitting.^24^

To map the stability of CeO_2_ surfaces under varying strains, we present an extensive modeling study on the interaction of water on CeO_2_ surface. We couple this with a thermodynamic strategy by generating phase diagrams to evaluate the strained structures under different temperature and pressures. This provides insights into strain effects in ultrathin nanoparticles (NPs), NPs supported on thin films, and core@shell structures, which will advance the design of more efficient strained nanocatalysts and nanozyme.

## Methodology

Density functional theory (DFT) spin polarized calculations were carried out using the Vienna *Ab initio* Simulation Package (VASP).^25^ The calculations used a plane wave basis set with a cutoff energy of 500 eV, and the core–valence interaction represented using the projector augmented wave (PAW) approach.^26^ The frozen core is [He] for O and [Xe] for Ce. The exchange–correlation functional was the GGA Perdew–Burke–Ernzerhof for solids (PBEsol),^27^ with the inclusion of the Hubbard correction parameter using the Dudarev methodology.^28^ A *U* = 5 eV was selected to aid localization of the 4f electrons as in previous studies.^26,29−34^

### Bulk CeO_2_

Bulk CeO_2_ was minimized with electronic and ionic criteria of 10^–6^ eV per atom and 10^–2^ eV Å^–1^. *K*-point sampling of the Brillouin zone uses the Γ-centered 5 × 5 × 5 *k*-mesh. The 4 unit (CeO_2_) minimized bulk cell gave a lattice constant of 5.497 Å, which is a known overestimation of the experimental value of 5.411 Å.^31^

### CeO_2_ Surfaces

The {100}, {111}, and {110} surfaces were generated using the METADISE code^35^ from the minimized bulk CeO_2_. The slab method was used so that the top and bottom of the configurations are identical. A vacuum gap of 15 Å was introduced perpendicular to the surface plane, which minimizes interactions between unit cells. The {100} and {111} slabs with (√2 × √2) and the {110} slab with (2 × √2) expansions of the primitive surface unit cell were investigated. The {100} and {110} surfaces were 7 layers (28 CeO_2_ units) and the {111} was 5 layers (20 CeO_2_ units). The dipole moment of the {100} surface is removed by transferring half of the oxygen from the top to the bottom of the slab resulting in a zero net dipole moment. The Brillouin zone was sampled using a Monkhorst–Pack Γ-centered 2 × 2 × 1 *k*-point grid with the third vector perpendicular to the surface plane. The criteria for electronic and ionic convergence were 10^–6^ eV per atom and 10^–2^ eV Å^–1^. When all attempts to stabilize specific configurations failed, they were run with lower electronic and ionic criteria of 10^–4^ eV per atom and 10^–1^ eV Å^–1^, respectively. These include: {100}S-25H_2_O-M (4 to 5%), {110}S-25H_2_O-M (4 to 5%), {111}S-25H_2_O-M (4 to 5%), {100}S-50H_2_O-D(−4 to −5%), {100}S-50H_2_O-M (1.5 to 5%), {100}S-75H_2_O-M (−5 to 5%), {100}S-100H_2_O-M (0.5 to 5%), {111}S-50H_2_O-M (2.5 to 5%), {111}S-75H_2_O-D (−3 to −5%), {111}S-75H_2_O-M (3 to 5%). Note that generally they are at very high or low strain, apart from {100}S-75H_2_O-M and {100}S-100H_2_O-M, where we noted that this was the only way to stabilize the molecular form of all water molecules adsorbed at the same time at the top and bottom of the slab.

### Molecule

The isolated H_2_O molecule was minimized using the Γ point in a cubic cell of 1000 Å^3^. Criteria for convergence were the same as for the surfaces.

### Water Adsorption on CeO_2_

Water was adsorbed dissociatively as hydroxyl groups and associatively as molecular H_2_O. There is no change in magnetization of Ce atoms in the slab upon adsorption of dissociative water. The water molecule was symmetrically adsorbed on both the top and bottom surfaces of the slab. Different water coverages were investigated (25, 50, 75%) up to the monolayer (100%) adsorption, which corresponds to one water molecule per surface CeO_2_ unit. There are a number of ways that water can be placed on the surface, but we have limited ours to structures that maximize the coordination between the adsorbates and the surface, and have been reported in the literature (all configurations studied are in Figures S1–S3).^31,36,37^

### Surface Lattice Strain

An isotropic biaxial strain is applied to the surface plane only. Strain values range from +5% (tensile) to −5% (compressive) in intervals of 0.5%.

### Surface Labeling

We use labels such as *X*%_{hkl}*S*-NH_2_O–*Y*, where *X*% is the strain in percentage, {*hkl*} is the Miller index of the surface, *S* stands for stoichiometric, *N* is the water coverage, and *Y* is *M* for molecular and *D* for dissociative water.

### Visualization and Imaging

Structures were visualized and drawn using VESTA.^38^

## Results and Discussion

### The Energetics of Bare Surfaces

The surface energy γ_Bare_ was calculated from the energy of a stoichiometric surface *E*_Slab_^Stoich,Bare^, the energy of the unstrained bulk with the same number of CeO_2_ units as the surface *E*_Bulk,CeO_2__^Stoich^, and the surface area *A* (2 is to account for the two symmetrical surfaces):
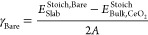
1

The surface energy (eq 1) defines the stability of a surface, and the values for the unstrained {100}S, {110}S, and {111}S stoichiometric CeO_2_ surfaces are in agreement with the literature, with the order of stability following {111}S < {110}S < {100}S,^20,25,29−31^ with {111} being the most stable, and {100} the least stable surfaces. Literature data for the surface energies are reported in Table 1.

**Table 1:** Surface Energy (J/m^2^) of the Stoichiometric {100}, {110}, and {111} CeO_2_ Surfaces Compared to Literature Values

methodology	{100}S (J/m^2^)	{110}S (J/m^2^)	{111}S (J/m^2^)
this work PBE+U = 5 eV	1.45	1.06	0.70
PBE+U = 6 eV^39^	1.63		
PBE^20^	1.704		
PBE^20^		1.214	0.834
PBE+U = 5 eV	1.44	1.06	0.71
PBE+U = 5 eV^30^	1.41	1.05	0.67
PW91+U = 7 eV^25^		0.96	0.60
PW91+U = 5 eV^40^	1.65	1.39	0.68
PW91+U = 5 eV^41^	1.41	1.01	0.68
LDA^41,42^		1.35	1.04
PW91+U = 5 eV	1.41	1.04	0.69
LDA-PW91^43^		1.35	1.0
GGA-PW91^43^		1.05	0.7
HF^43^		2.11	1.31
PBE+U = 5 eV	1.45	1.06	0.70
PBE+U = 5 eV^26^	1.45	1.06	0.70
ML TU-TILD^a^ ^45^		1.19	0.78

a TU-TILD is combined with machine learning (ML) potentials and trained on results of ab initio molecular dynamics (AIMD).

**Figure 1:**
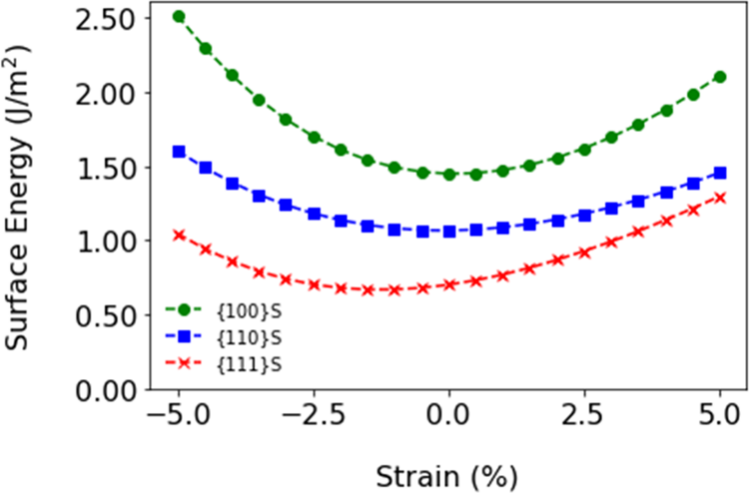
Surface energy of stoichiometric CeO_2_ surfaces under compressive (negative) and tensile (positive) strains.

The order of stability of strained and unstrained surfaces remains the same (Figure 1). Both tensile and compressive strain destabilize the surfaces, as they reduce and increase the length of Ce–O bonds, resulting in a weaker and stronger overlap of O 2p and Ce 5d/4f states, respectively.^46^ It is interesting that the difference in surface energy between {110}S and {111}S decreases significantly as the tensile strain increases, while the difference in the energy among the three surfaces increases as compressive strain increases (Figure 1). Upon compressive strain between −2 and −0.5%, the surface energies of the strained {111} surface are lower than the unstrained {111} surface. However, these differences are relatively small of the order of 0.02–0.03 Jm^2–^, which correspond to a 3–4.6% error on our energies. This effect is due to the small size of the {111} slab model used in this study with thickness of 5 surface layers.

### The Energetics of Water Adsorption

The adsorption energy of water (eq 2) on stoichiometric surfaces is calculated from the energy of the stoichiometric surfaces with adsorbed water *E*_Slab_^Stoich,Ads^, the energy of a stoichiometric bare surface *E*_Slab_^Stoich,Bare^, and the energy of *n*_H_2_O_ water molecules *E*_H_2_O_ (where *n* = 1, 2, 3, 4 correspond to 25, 50, 75, and 100% water coverage on each side of the slab, and 2 is to account for the two symmetrical surfaces):
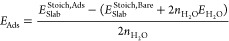
2

The adsorption energies for 25% water coverage of dissociative (−1.54 eV {100} > −1.06 eV {110} > −0.5 eV {111}), and molecular (−0.90 eV {100} > −0.77 eV {110} > −0.53 eV {111}) water adsorption on the stoichiometric surfaces are in line with the literature.^29,31,44,47^ Literature data for the adsorption energies are reported in Table 2. Of note, the adsorption energies of the 0%_{111}S-25H_2_O-M (−0.53 eV) and 0%_{111}S-25H_2_O-D (−0.50 eV) compare well with the experimental values of (−)0.53 eV (at 0.2 ML) and (−)0.61 eV (unknown coverage) measured using temperature-programmed desorption (TPD) experiments.

**Table 2:** Adsorption Energy of Water (eV) for Both Molecular and Dissociatively Adsorbed Water on Stoichiometric CeO_2_ Surfaces at Different Coverages in H_2_O/nm^2^ and ML (Monolayer) in Brackets

surface label	methodology	adsorption energy (eV)	coverage H_2_O/nm^2^ (ML)
0%_{111}S-NH_2_O-M^29^	PBE+U = 5 eV	–0.58/–0.56	1.28 (0.17)
0%_{111}S-NH_2_O-M	this work, PBE+U = 5 eV	–0.53	1.90 (0.25)
0%_{111}S-NH_2_O-M^48^	PW91+U = 5 eV	–0.35	- (0.25)
0%_{111}S-NH_2_O-M^44^	PBE+U = 5 eV	–0.5	1.90 (0.25)
0%_{111}S-NH_2_O-M^40^	PW91+U = 5 eV	–0.51	- (0.25)
0%_{111}S-NH_2_O-M^49^	PBE	–0.49	- (0.25)
0%_{111}S-NH_2_O-M	this work, PBE+U = 5 eV	–0.59	3.81 (0.5)
0%_{111}S-NH_2_O-M^25^	PW91+U = 7 eV	–0.51	- (0.5)
0%_{111}S-NH_2_O-M^29^	PBE+U = 5 eV	–0.60	3.83 (0.5)
0%_{111}S-NH_2_O-M	this work, PBE+U = 5 eV	–0.56	7.64 (1)
0%_{111}S-NH_2_O-M^29^	PBE+U = 5 eV	–0.57	7.67 (1)
0%_{111}S-NH_2_O-D^31^	PBE+U = 5 eV	–0.6	1.27 (0.16)
0%_{111}S-NH_2_O-D^29^	PBE+U = 5 eV	–0.59	1.28 (0.17)
0%_{111}S-NH_2_O-D	this work, PBE+U = 5 eV	–0.50	1.90 (0.25)
0%_{111}S-NH_2_O-D^50^	PBE+U = 5 eV	–0.57	- (0.25)
0%_{111}S-NH_2_O-D^48^	PW91+U = 5 eV	–0.65	- (0.25)
0%_{111}S-NH_2_O-D^44^	PBE+U = 5 eV	–0.5	1.90 (0.25)
0%_{111}S-NH_2_O-D^49^	PBE	–0.65	- (0.25)
0%_{111}S-NH_2_O-D^31^	PBE+U = 5 eV	–0.7	2.54 (0.33)
0%_{111}S-NH_2_O-D	this work, PBE+U = 5 eV	–0.41	3.81 (0.5)
0%_{111}S-NH_2_O-D^51^	PBE	–0.58	- (0.5)
0%_{111}S-NH_2_O-D^31^	PBE+U = 5 eV	–0.58	3.81 (0.5)
0%_{111}S-NH_2_O-D^31^	PBE+U = 5 eV	–0.56	5.09 (0.66)
0%_{111}S-NH_2_O-D	this work, PBE+U = 5 eV	–0.13	7.64 (1)
0%_{111}S-NH_2_O-D^29^	PBE+U = 5 eV	–0.15	7.67 (1)
0%_{111}S-NH_2_O-D^51^	PBE	–0.55	- (1)
0%_{110}S-NH_2_O-M	this work, PBE+U = 5 eV	–0.76	1.16 (0.25)
0%_{110}S-NH_2_O-M^44^	PBE+U = 5 eV	–0.8	1.17 (0.25)
0%_{110}S-NH_2_O-M^29^	PBE+U = 5 eV	–0.85	1.17 (0.25)
0%_{110}S-NH_2_O-M	this work, PBE+U = 5 eV	–0.75	2.33 (0.5)
0%_{110}S-NH_2_O-M^29^	PBE+U = 5 eV	–0.76	4.69 (0.5)
0%_{110}S-NH_2_O-D	this work, PBE+U = 5 eV	–1.06	1.16 (0.25)
0%_{110}S-NH_2_O-D^44^	PBE+U = 5 eV	–1	1.17 (0.25)
0%_{110}S-NH_2_O-D^31^	PBE+U = 5 eV	–1.15	1.16 (0.25)
0%_{110}S-NH_2_O-D^29^	PBE+U = 5 eV	–1.12	1.17 (0.25)
0%_{110}S-NH_2_O-D	this work, PBE+U = 5 eV	–1.08	2.33 (0.5)
0%_{110}S-NH_2_O-D^31^	PBE+U = 5 eV	–1.2	2.3 (0.5)
0%_{110}S-NH_2_O-D	this work, PBE+U = 5 eV	–1.0	3.5 (0.75)
0%_{110}S-NH_2_O-D^31^	PBE+U = 5 eV	–1.1	3.5 (0.75)
0%_{110}S-NH_2_O-D^29^	PBE+U = 5 eV	–1.00	4.69 (0.5)
0%_{110}S-NH_2_O-D	this work, PBE+U = 5 eV	–0.97	4.67 (1)
0%_{110}S-NH_2_O-D^29^	PBE+U = 5 eV	–0.21	9.39 (1)
0%_{100}S-NH_2_O-M	this work, PBE+U = 5 eV	–0.90	1.65 (0.25)
0%_{100}S-NH_2_O-M^29^	PBE+U = 5 eV	–1.00	1.66 (0.25)
0%_{100}S-NH_2_O-M^44^	PBE+U = 5 eV	–0.82	1.65 (0.25)
0%_{100}S-NH_2_O-M	this work, PBE+U = 5 eV	–0.84	6.61 (1)
0%_{100}S-NH_2_O-M^29^	PBE+U = 5 eV	–0.89	6.64 (1)
0%_{100}S-NH_2_O-D	this work, PBE+U = 5 eV	–1.54	1.65 (0.25)
0%_{100}S-NH_2_O-D^29^	PBE+U = 5 eV	–1.57	1.66 (0.25)
0%_{100}S-NH_2_O-D^31^	PBE+U = 5 eV	–1.6	1.65 (0.25)
0%_{100}S-NH_2_O-D^44^	PBE+U = 5 eV	–1.5	1.65 (0.25)
0%_{100}S-NH_2_O-D	this work, PBE+U = 5 eV	–1.41	3.30 (0.5)
0%_{100}S-NH_2_O-D^29^	PBE+U = 5 eV	–1.73/–0.87	3.32 (0.5)
0%_{100}S-NH_2_O-D^31^	PBE+U = 5 eV	–1.3	3.5 (0.5)
0%_{100}S-NH_2_O-D	this work, PBE+U = 5 eV	–1.46	4.96 (0.75)
0%_{100}S-NH_2_O-D^31^	PBE+U = 5 eV	–1	4.96 (0.75)
0%_{100}S-NH_2_O-D	this work, PBE+U = 5 eV	–1.42	6.61 (1)
0%_{100}S-NH_2_O-D^29^	PBE+U = 5 eV	–0.89	6.64 (1)

**Figure 2:**
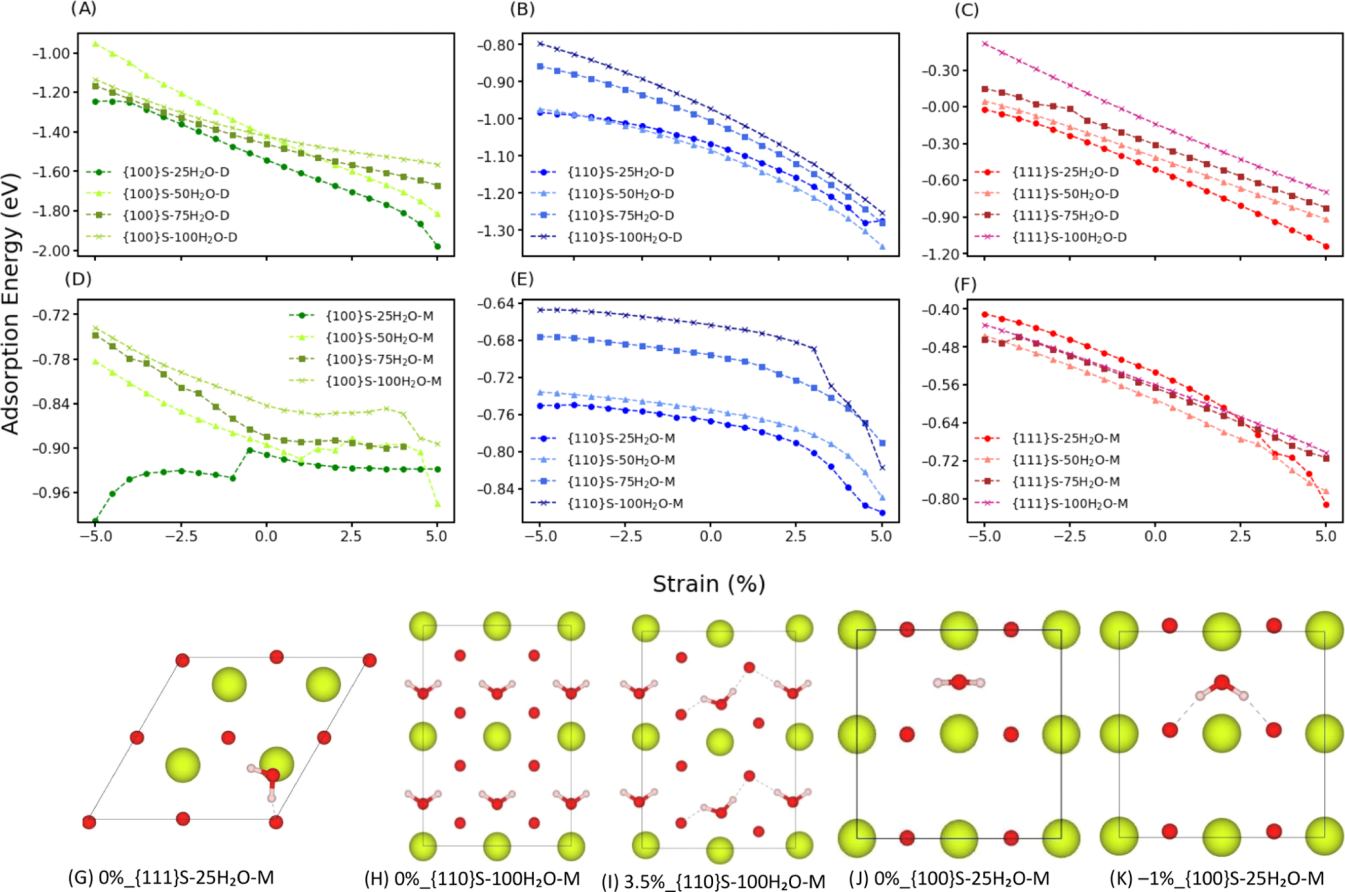
Adsorption energy of water on CeO_2_ surfaces as a function of strain and water coverages (25–100%): dissociative water on the (A) {100}, (B) {110}, and (C) {111} surfaces, and molecular water on the (D) {100}, (E) {110}, and (F) {111} surfaces. The structure of adsorbed molecular water on (G) {111}, (H, I) {110}, and (J, K) {100} unstrained and strained surfaces.

The water adsorption on CeO_2_ surfaces from 25% coverage to the monolayer adsorption as a function of compressive and tensile strain is presented in Figure 2A–F. Applied strain does not change the order of stability of adsorbed water on the stoichiometric surfaces. This is reflected in the hydrogen bonding network, which does not change drastically with strain (Figures S4–S5). Compressive strain destabilizes while tensile strain stabilizes water adsorption for all coverages of both the molecular and dissociative adsorptions. Increasing water coverage sees a destabilization of the dissociative water adsorption, which is also seen for the molecular adsorption of water on the {100} and {110} surfaces. Unlike the other surfaces, molecular adsorption is stabilized by an increase in water coverage for the {111} surface. However, the change in the adsorption energy of molecular water on the {111}S is quite small upon increasing coverage (0.02 eV from 25% to 100% water coverage), which is also supported by the literature.^29,49^ Perhaps the most dramatic change is seen for the dissociative adsorption of water on the {111} surface, as under compressive strain the adsorption energy becomes positive, which is more pronounced as the water coverage increases.

Here, we only present the salient structural features of the water adsorption (Figure 2G–K), leaving detailed schematics of all adsorption geometries in Figures S1–S3. The adsorption configurations of dissociative water on the three surfaces of ceria do not change radically as a function of water coverage and strain. The same applies to the adsorption of molecular water on the {111} surface (Figure 2G). A reduced H bonding network would normally see a destabilization in the adsorption energy, but strain induces a reconstruction of the surface layers for the {110} surface, which is most likely the underlying cause of the large stabilization of the adsorption of molecular water on the {110} surface above 2.5% tensile strain (Figure 2H vs I). Although this reconstruction on the {110} surface occurs for all values of tensile strain, this appears to affect the adsorption of molecular water more, and to a lesser extent the adsorption of dissociative water. We also observe a breakdown in the adsorption trend for values of compressive strain below −0.5% for the adsorption of molecular water at 25% water coverage on the {100} surface. However, visual inspection reveals that this is not due to a surface restructuring, but a change in the adsorption configuration of molecular water molecules on the {100} surface (from flat to tilted, Figure 2J vs K) as reflected also in the hydrogen bonding network (Figure S4).

### Temperature of Desorption

To compare the adsorption of water on the strained ceria surfaces, we calculate the temperature of desorption (*T*_D_) using the approach implemented in Surfinpy.^52,53^ This approach has been used successfully in previous studies.^29−31,44,54,55^*T*_D_ is the temperature at which water desorbs from the surface of ceria at a specific partial pressure of water, thus describing the phase boundary between two phases: the bare surface and the surface with adsorbed water.

The surface energy of surfaces with adsorbed water (eq 3) can be calculated from the surface energy of bare surfaces (eq 1), the water coverage , the number of adsorbed water molecules *n*_H_2_O_, the surface area *A*, gas constant *R*, temperature *T*, and the partial pressures of water chosen *p*_H_2_O_ and in the standard state *p*^o^:
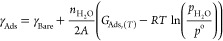
3where the adsorption energy of water as a function of temperature (eq 4) is
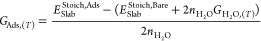
4and the energy of water including temperature effects, *G*_H_2_O_,_(*T*)_ (eq 5), is calculated from the experimental entropy of gaseous water in the standard state *S*_(T)_:^56^
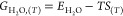
5Future developments would benefit from calculating the vibrational frequency contributions of the solid phases (*E*_Slab_^Stoich,Ads^ and *E*_Slab_^Stoich,Bare^) to obtain a more accurate estimate of the free energy change. Our approach also does not include any kinetic effects due to the dissociation of water and this again should be in interesting topic to develop further.

**Figure 3:**
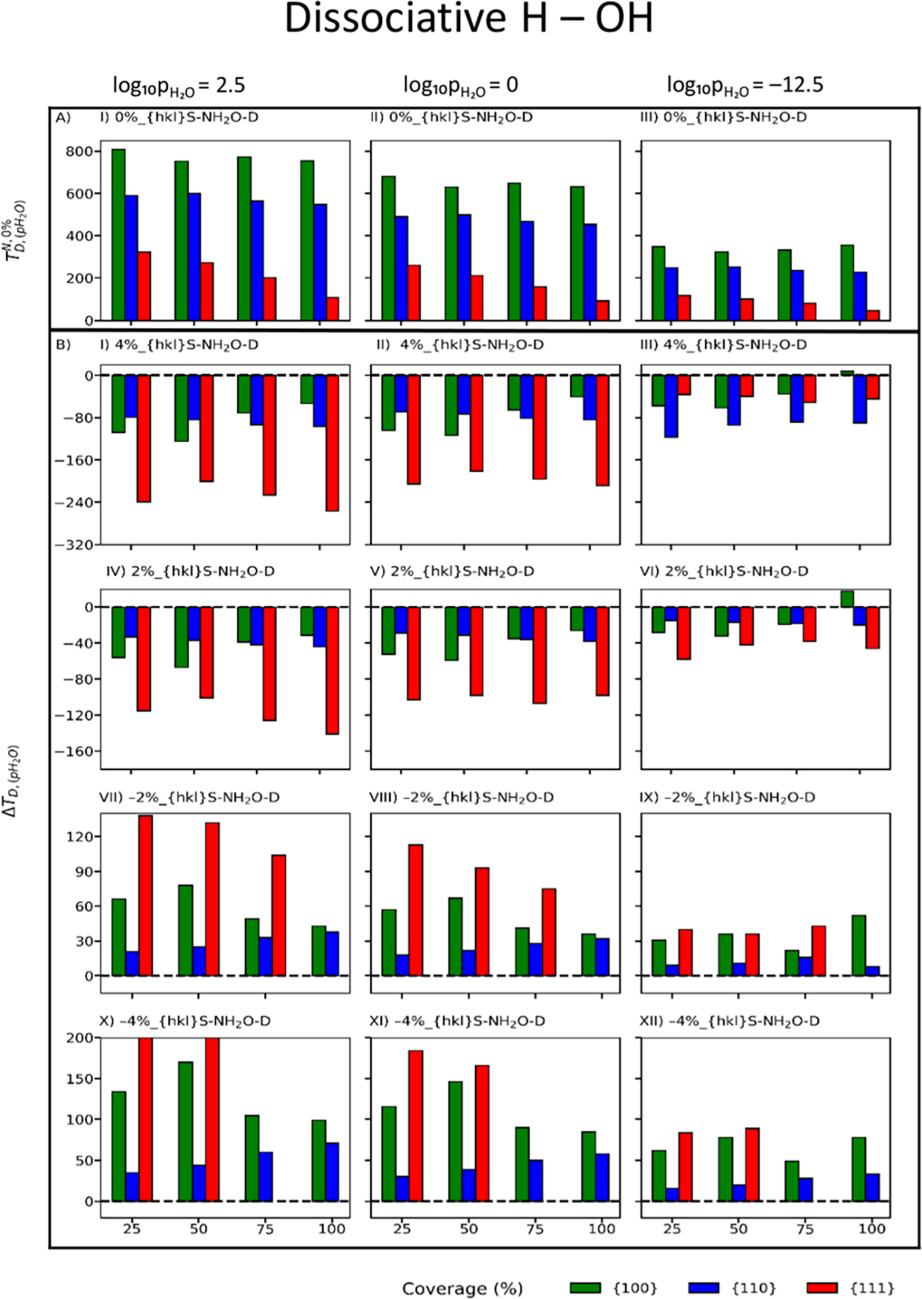
Temperature of desorption of dissociative (A) adsorbed water on the unstrained surfaces of CeO_2_. Change in the temperature of desorption with respect to the unstrained surface (Δ*T*_*D*,(*p*H_2_O)_) of dissociative (B) adsorbed water on the strained surfaces of CeO_2_ at compressive (−4 and −2%) and tensile (4 and 2%) strains. Red, blue and green represent the {111}, {110}, and {100} CeO_2_ surfaces, respectively.

**Figure 4:**
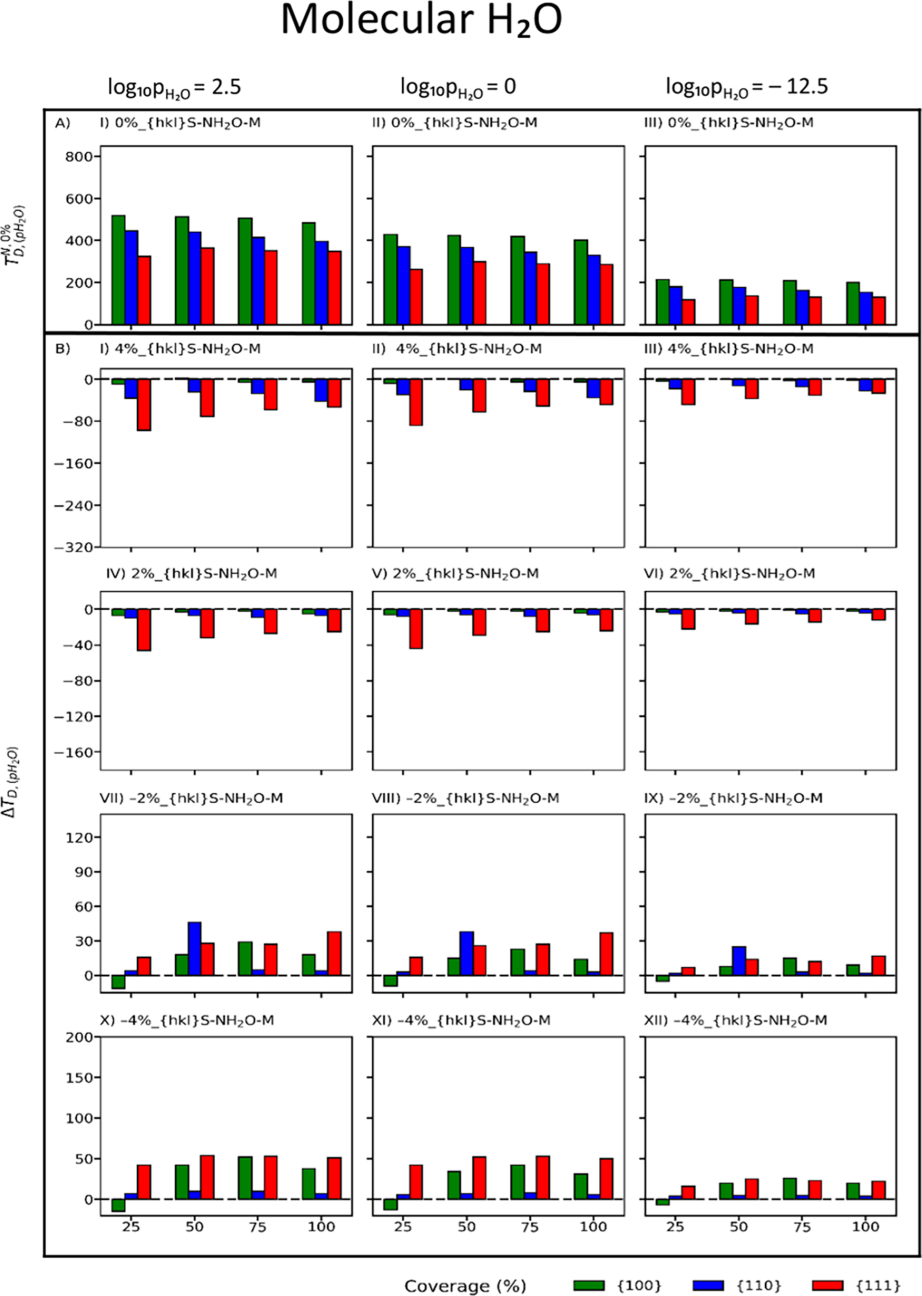
Temperature of desorption of molecular (A) adsorbed water on the unstrained surfaces of CeO_2_. Change in the temperature of desorption with respect to the unstrained surface (Δ*T*_*D*,(*p*H_2_O)_) of molecular (B) adsorbed water on the strained surfaces of CeO_2_ at compressive (−4 and −2%) and tensile (4 and 2%) strains. Red, blue and green represent the {111}, {110}, and {100} CeO_2_ surfaces, respectively.

Figures 3A and 4A map the desorption temperatures of dissociative and molecular adsorbed water on unstrained CeO_2_ surfaces at different water coverage for selected partial pressures of water. Molecular water has a lower *T*_D_ than dissociative water, with water desorbing at lower temperature on the {111} followed by the {110} and the {100} surfaces at all partial pressures of water. *T*_D_ decreases with increasing coverage as the adsorption energy of water becomes less negative as coverage increases (Figure 2). This is in line with previous literature.^29,31^

Experimental measurements have revealed that a mixture of molecular and dissociative water can adsorb on the stoichiometric {111} and {100} surfaces of ceria.^57−64^ Experimental data on the {110} CeO_2_ surface is not available.

Little experimental data is available for the oxidized {100} CeO_2_ surface, where water desorption has been shown to extend from near 200 K to beyond 500 K, with three peaks above 200, 300, and 400 K, on the {100} ceria surface indicating that water predominantly dissociated on this surface.^57^ Our data for molecular and dissociatively adsorbed water shows temperatures of desorption in the range 222–238 and 356–393 K, respectively depending on coverage and assuming a partial pressure of 10^–10^ bar. The peak above 200 K is assigned to the presence of molecular adsorbed water, while the remaining peaks are related to strongly adsorbed hydroxyl groups.^57^

Most studies have been performed on the most stable {111} ceria surface. According to Temperature-Programmed Desorption (TPD) spectra, H_2_O desorbs as a sharp feature around 200 K on the oxidized {111} ceria surface,^57,59−61^ while hydroxyl groups mostly disappear below 250 K.^57−59,61^ In the following text we have discussed relevant data where comparison could be done under certain assumptions.

A range of 170–185 K desorption temperatures (measured at 10^–10^ bar) was reported for the single water layer on the oxidized {111} CeO_2_ surface grown on the Cu(111) substrate, and a water desorption peak around 200 K (starting from 140–150 K) was measured at 10^–10^ bar on the oxidized {111} CeO_2_ surface grown on Ru(0001).^58^ Furthermore, D_2_O was also found to desorb (using TDP at 10^–10^ bar) in three states with temperatures of 152, 200, and 275 K, representing respectively multilayer D_2_O, weakly bound surface D_2_O, and hydroxyl recombination.^63^ These experimental values are higher than our calculated temperature of 147 K for molecular water (at 10^–10^ bar) at the monolayer adsorption. In the range 130–200 K, a much higher concentration of molecular water was found compared to dissociatively adsorbed water,^58^ which would support the computational finding that at the monolayer saturation molecularly adsorbed water has a much higher stability then dissociatively adsorbed water (Figure 2). However, experimentally chemisorbed water or multilayer/physisorbed water may coexist up to 150–170 K.^65^

Water desorption peaks were also measured in the range 195–320 K (at 10^–12^ bar) depending on water coverage on the oxidized {111} CeO_2_ surface grown on the YSZ(111) substrate.^60^ The 320 K is mostly the background adsorbed water (0.04–0.07 ML). As the water coverage is increased from 0.2 to 0.9 ML, the water desorption peak shifts from 265 to 200 K. This behavior was conceptualized considering the hydrogen bond network and water molecules being forced to reduce the number of hydrogen bonds to the surface when their concentration increases. The experimental values^60^ are much higher than our calculated temperature of 119, 137, 131, 130 K for molecular water (at 10^–12^ bar) at 0.25, 0.5, 0.75, and 1 ML but much lower than 350 K at 0.25 ML calculated including the oxygen chemical potential.^49^ We need to go to partial pressures of water of 10^–5^ bar to see temperature of desorption similar in magnitude to the experiments (171, 198, 190, and 188 K at 0.25, 0.5, 0.75, and 1 ML).

The discrepancies between the calculated and experimental temperatures of desorption are symptomatic of an experimental complexity that is yet to be considered in the calculations. However, considering the simple models used in the DFT calculations, these can provide some additional knowledge. Future studies should include mixed states of adsorbed water and surface structural features.

For each surface, the effect of strain can be better visualized as a change in desorption temperature (eq 6) with respect to the *T*_D_ of the unstrained *T*_*D*,(*p*H_2_O)_^*N*,0%^ and strained *T*_*D*,(*p*H_2_*O*)_^*N*,Strain%^ surface at a specific partial pressure (*p*_H_2_O_):
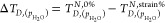
6A Δ*T*_*D*,(_*p*_H_2_O)_ < 0 indicates that the stability of the adsorption of water on strained surfaces is higher than that of unstrained surfaces, as *T*_*D*,(*p*H_2_O)_^*N*,Strain%^ > *T*_*D*,(*p*H_2_O)_^*N*,0%^. The opposite occurs if Δ*T*_*D*,(*p*H_2_O)_ > 0. All temperatures of desorption for all configurations are provided in Figures S6–S7.

Δ*T*_*D*,(*p*H_2_O)_ is much greater in magnitude for dissociatively than for molecularly adsorbed water. Irrespective of dissociative and molecular water adsorption, tensile strain sees negative changes (Δ*T*_*D*,(*p*H_2_O)_ < 0) in the desorption temperatures with a few exceptions (4%_{100}S-100H_2_O-D, 2%_{100}S-100H_2_O-D), and Δ*T*_*D*,(*p*H_2_O)_ also becomes more negative as tensile strain increases (Figures 3B(I–VI) and 4B(I–VI)). Conversely, compressive strain sees positive changes (Δ*T*_*D*,(*p*H_2_O)_ > 0) in the desorption temperatures with a few exceptions (2%_{100}S-25H_2_O-D, −2%_{100}S-25H_2_O-D, −2%_{100}S-25H_2_O-D, −4%_{100}S-25H_2_O-D, −4%_{100}S-25H_2_O-D, −4%_{100}S-25H_2_O-D), and Δ*T*_*D*,(*p*H_2_O)_ also becomes more positive as compressive strain increases (Figures 3B(VII–XII) and 4B(VII–XII)). Finally, on strained surfaces, as the partial pressure of water decreases, Δ*T*_*D*,(*p*H_2_O)_ becomes less negative and less positive for tensile and compressive strains, respectively.

### Morphology of Strained Cerium Dioxide Nanoobjects

The prediction of the shape of nanoobjects, either nanoparticles or nanograins, is important as it will define the thermodynamic shape of the constituents of the material. If the nanoobject is a nanoparticle, then this can be grown on a substrate or be part of a core@shell structure, where the mismatch between the lattices of the different materials will define the strain imposed on the nanoparticle and thus its thermodynamic morphology. If the nanoobject is a nanograin, then this would be part of a poly nanocrystalline material and, as such, be strained by other differently oriented nanograins of the same material or of different materials in composites.

**Figure 5:**
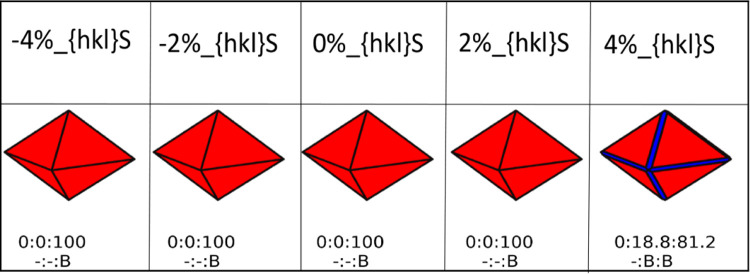
Shapes of CeO_2_ bare nanoparticles. Red, blue and green represent the {111}, {110}, and {100} CeO_2_ surfaces, respectively. The shapes of the nanoparticles are generated at a water partial pressure of 1 bar and 298 K. Each shape is characterized by a ratio of surfaces defined in numerical percentage ({100}:{110}:{111}). The label B defines a bare surface.

The shape of ceria nanoobjects as a function of strain, temperature and partial pressure are generated using the Wulff construction^66−68^ and follow a well-established methodology.^30,31^ To generate the morphologies, the surface area phase diagrams representing surface composition alongside the ratio of the three surfaces are also needed and presented in the Supporting Information (Figures S8–S13). For each morphology presented in Figures 4 and 5, we also report the ratio of the surfaces expressed by the nanoparticle, i.e., {100}:{110}:{111}. It is this ratio determines the nanoparticle morphology. Each surface expressed by the nanoobject also has a different composition as it could be hydrated or bare depending on the external conditions.

When we only consider bare surfaces (Figure 5), the {111} surface dominates all morphologies for CeNPs, at any compressive and tensile strain.

**Figure 6:**
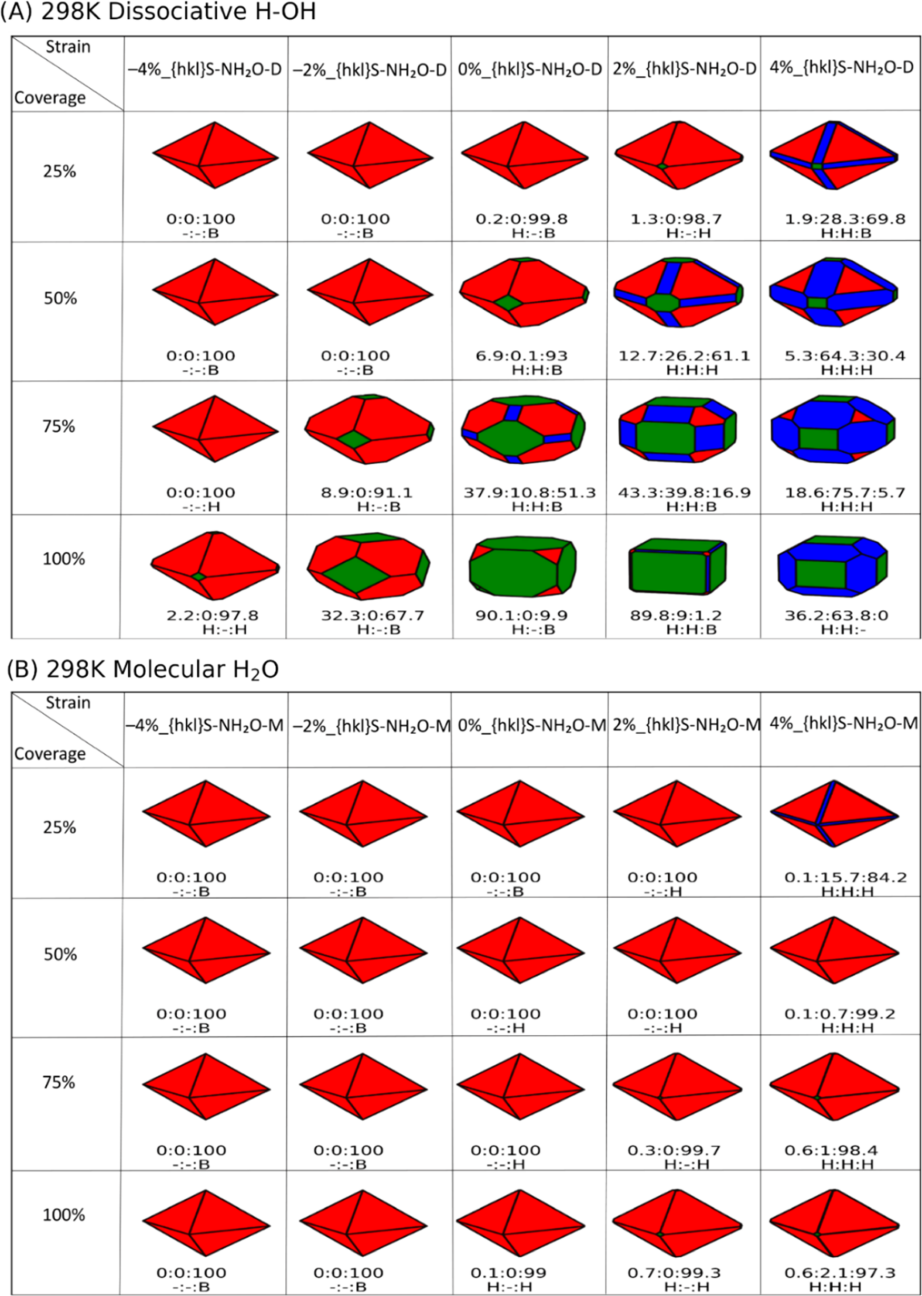
Shapes of CeO_2_ nanoparticles in the presence of dissociative (A) and molecular (B) water. Red, blue and green represent the {111}, {110}, and {100} CeO_2_ surfaces, respectively. The shapes of the nanoparticles are generated at a water partial pressure of 1 bar and 298 K. Each shape is characterized by a ratio of surfaces defined in numerical percentage ({100}:{110}:{111}). There is also a set of labels, H (hydrated surface) and B (bare surface), which determine whether the corresponding surface has water adsorbed on it or not.

For dissociative water adsorption (Figure 6A), increasing water coverage at constant strain primarily stabilizes the {100} and {110} surfaces, particularly for tensile strain: the octahedral shape leads the way to the formation of truncated octahedral, cuboidal, and truncated cuboidal morphologies at higher water coverage and strain. Under standard temperature and pressure conditions, at all strains the {110} and {100} surfaces are hydrated, while the {111} surface transitions between hydrated and bare at higher strain depending on higher water coverage.

For molecularly adsorbed water (Figure 6B), an octahedral nanoobject expressing the {111} surface is the predominant morphology for all water coverages for all strain values. There is minimal appearance of the {110} surface as an edge and the {100} surface as a truncated corner, under tensile strain and it is more accentuated at lower water coverage. Under standard temperature and pressure conditions, at all strains the {110} and {100} surfaces are hydrated, whereas unlike compressive strain, tensile strain stabilizes the hydration of the {111} surfaces. As the temperature increases from 298 to 673 K (Figures S14–S17), we see an increase in the {110} surface in the morphology of CeNPs when molecular water is adsorbed, but this is not sufficient to significantly deform the octahedral shape of the nanoparticles. When water is adsorbed dissociatively, and temperature is increased, we observed the opposite behavior with the disappearance of the {110} and {100} surfaces (which would otherwise stabilize truncated octahedral morphologies), while the {111} surface becomes predominant, favoring a more pristine octahedral shape.

**Figure 7:**
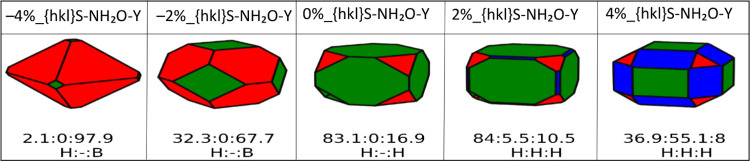
Shapes of CeO_2_ nanoparticles at 298 K and 1 bar partial pressure of water, considering only the most stable phases within a constant value of strain, at all water coverages and independently on the dissociative or molecular water adsorption. Each shape is characterized by a ratio of surfaces defined in numerical percentage ({100}:{110}:{111}). There is also a set of labels, H (hydrated surface) and B (bare surface), which determine whether the corresponding surface has water adsorbed on it or not. Red, blue and green represent the {111}, {110}, and {100} CeO_2_ surfaces, respectively.

For completeness, we have generated morphologies at 298 K and 1 bar partial pressure of water (Figure 7), considering only the most stable phases within a constant value of strain, at all water coverages and independently on the dissociative or molecular water adsorption (i.e., X%_{100}S-100H_2_O-D, X%_{110}S-100H_2_O-D, and X%_{111}S-100H_2_O-M). These nanoparticles are similar to the shapes of those constructed using only surfaces adsorbed with dissociative water molecules at the monolayer adsorption (100% water coverage, Figure 6A).

Controlling the shape of nanoparticles is important to tune catalytic activity. Cuboidal NPs mostly expressing the {100} surface^69^ display enhanced CO catalytic oxidation compared with truncated octahedral NPs exposing both the {111} and {100} surfaces.^70^ As water facilitates the CO oxidation process,^71^ tensile strain and a full monolayer of water would be needed to stabilize cuboidal nanoceria as shown in Figure 6A.

Experimentally, many factors have been shown to affect the shape of nanoceria, including the concentration of alkaline solutions,^72^ the presence of precipitating agents,^73^ capping agents,^74^ the solution pH,^75^ the synthesis temperature^76,77^ and time,^78^ and thermal aging.^79^ Strain is another factor but is as of yet less explored since it is more complicated to control this experimentally. Computationally, of course, this is not the case so modeling can be used as an explorative tool.

The use of strain to tune the morphology of nanoparticles is indeed less common,^80,81^ with few studies on ceria NPs. Imposing an intrinsic surface strain by crossing the nanoscale can be an effective strategy to regulate catalytic (nanozymatic) activity. This has been shown in ultrathin ∼1.2 nm CeO_2_ nanoplates that experience an intrinsic surface tensile strain (3.0% in plane/10.0% out of plane), which ultimately induces a tetragonal distortion and an increase in the covalency of the Ce–O bonds, leading to a ∼2.6-fold increase in superoxide dismutase (SOD) mimetic activity compared with unstrained ceria nanoparticles.^12^ Although, our results did not display nanoplates, this would depend on synthesis conditions and there will be kinetic effects that we cannot yet simulate. Whereas phase transitions are known to occur in CeNPs under strain,^82^ voids of geometrical shapes,^83,84^ and faceting^85,86^ have still to be related to the presence of localized surface strain.

Lattice mismatch induced strain is at the core of core@shell structures and supported films. Whereas lattice strain can be easily calculated in supported films, it is more complicated when it comes to core–shell structures. Crystalline supported nanofilms CeO_2_(100)/STO(001) and CeO_2_(100)/YSZ(001) experience tensile (2.1%) and compressive (−5.6%) strains respectively, which are linked to an increase in the concentrations of Ce^3+^ (up to 4-fold).^14^ On the other hand, a precise measure of the strain exerted by the lattice mismatch in core@shell nanostructures is difficult because of the curvature of the nanoobject, which makes it even more complicated to evaluate the effect of strain on the exerted activity.

However, it is also complicated to decouple the intricate interplay established between the lattice mismatch (and thus strain) strain and the nature of the interaction between the different materials in the core@shell structures, and whether these interactions may enhance activity of such nanostructures. Although there is no information on the strain exerted by the different materials, there are some examples to cite where core@shell structures can improve the photocatalytic reduction of CO_2_ (spherical CdS@CeO_2_),^87^ the photocatalytic water splitting (rod-like TiO_2_@CeO_2_),^88^ and the chemoselective reduction of nitro compounds (spherical AgNP@CeO_2_).^89^

## Conclusions

We have demonstrated that modeling based on density functional theory can aid the interpretation of strain effects in nanoceria. By evaluating the adsorption free energies for water adsorbed on strained surfaces of ceria, we can implement a thermodynamic strategy to evaluate the morphology of particles (whether present as nanoparticles or nanograins) as a function of variables, such as temperature and partial pressure of water, that can be controlled experimentally.

Independent of tensile or compressive strain applied to the surface, the stability of low Miller index surfaces follows {111} > {110} > {100}, which is the same as unstrained surfaces. Compressive strain destabilizes the adsorption of water at different coverages, while tensile strain stabilizes it. We see drastic stabilization effects when the {110} surface structure restructures significantly under tensile strain.

The thermodynamic strategy implemented can aid the evaluation of the temperature of desorption of water on strained surfaces. We see that tensile strain increases the temperature of desorption while compressive strain has the opposite effect. We found that there is a thermodynamic driving force toward octahedral morphologies under all strains when molecular water is adsorbed on the surfaces of nanoceria. However, at high partial pressure of water and tensile strains, cuboidal nanoceria can be stabilized, if dissociative water is present at its surfaces.

Our atomistic modeling has allowed us to quantify strain effects related to nanoceria and its interaction with molecular and dissociative water. As we have only considered cases where water is separately adsorbed molecularly and dissociatively, future research should develop toward more complicated mixed phases. Such an approach can be used widely for the prediction of strained particle morphologies of materials and minerals in relevant environmental conditions.

## Data Availability

Raw data are available at https://doi.org/10.17632/3hwx68h2ny.

## Supporting Information Available

Adsorption configurations of water on strained ceria surfaces (Figures S1–S3), the hydrogen bond network between adsorbed water and ceria surfaces (Figures S4–S5), temperature of desorption of water on ceria surfaces (Figures S6–S7), “surface area” phase diagrams (Figures S8–S13), and predicted ceria particle morphology (Figures S14–S17) (PDF)
