# Supplementary material for: Strain Effects on the Adsorption of Water on Cerium Dioxide Surfaces and Nanoparticles: A Modeling Outlook
Source: J Phys Chem C Nanomater Interfaces. 2024 Oct 17;128(43):18451–64. doi: 10.1021/acs.jpcc.4c04172 (PMC11533200; doi:10.1021/acs.jpcc.4c04172)
Supplement: Supplementary file 1 — jp4c04172_si_001.pdf [file jp4c04172_si_001.pdf]

Supporting Information

Strain Effects on the Adsorption of Water on Cerium Dioxide Surfaces and Nanoparticles: A Modelling Outlook

Sidra Munir*[1], Khoa Minh Ta[1], Thomas Smith[1], Lisa J. Gillie[1], David J. Cooke[1], Stephen C. Parker,[2] and Marco Molinari*[1]
[1]Department of Physical and Life Sciences, University of Huddersfield, Queensgate, Huddersfield HD1 3DH, UK
[2]Department of Chemistry, University of Bath, Claverton Down, Bath BA2 7AY, UK
*Corresponding authors: sidra.munir@hud.ac.uk, m.molinari@hud.ac.uk

Section S1. Simulated structures for stoichiometric {100}, {110} and {111} ceria surfaces.

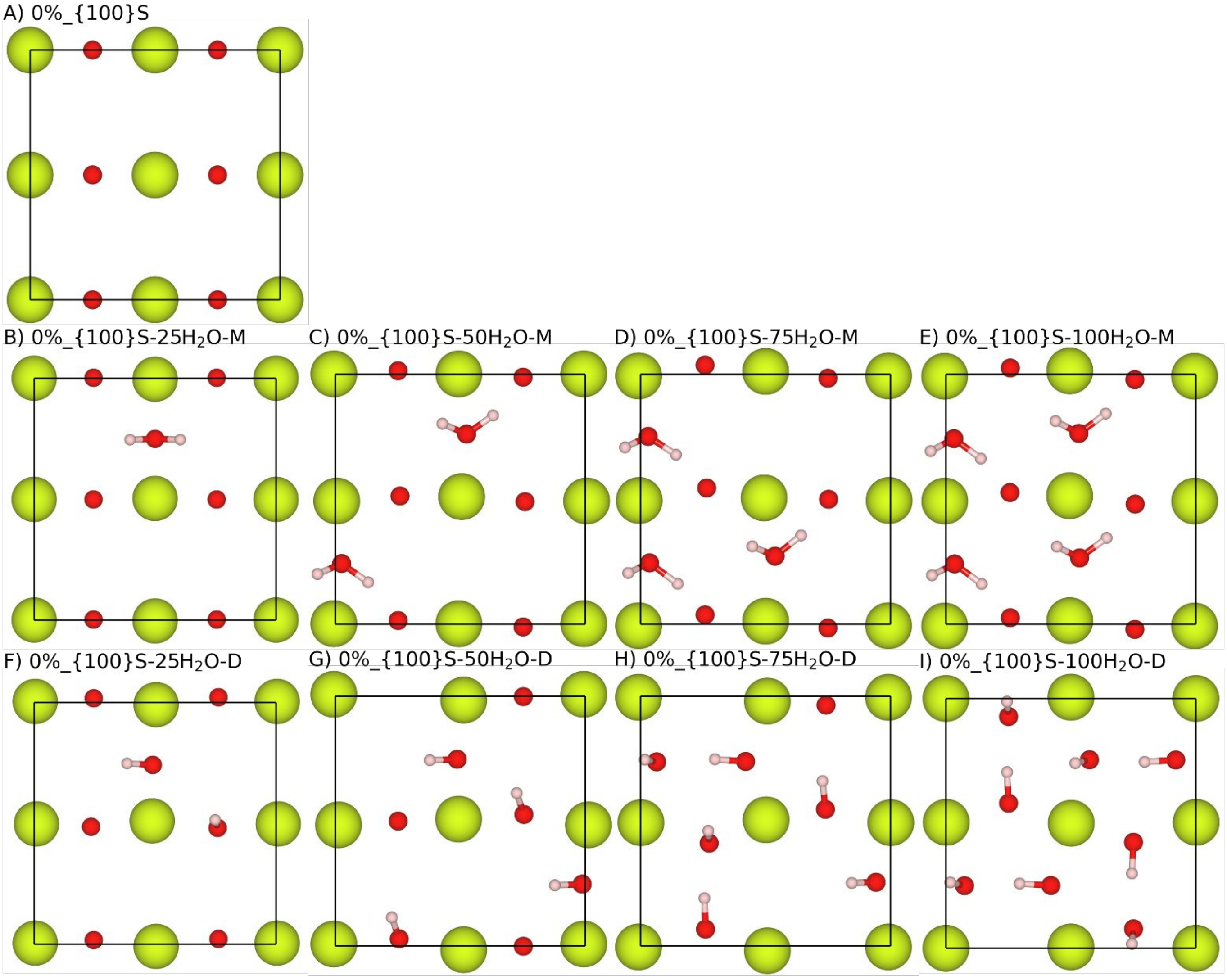

Figure S1. (A-I) Top view of the {100} CeO_2 configurations with adsorbed water.

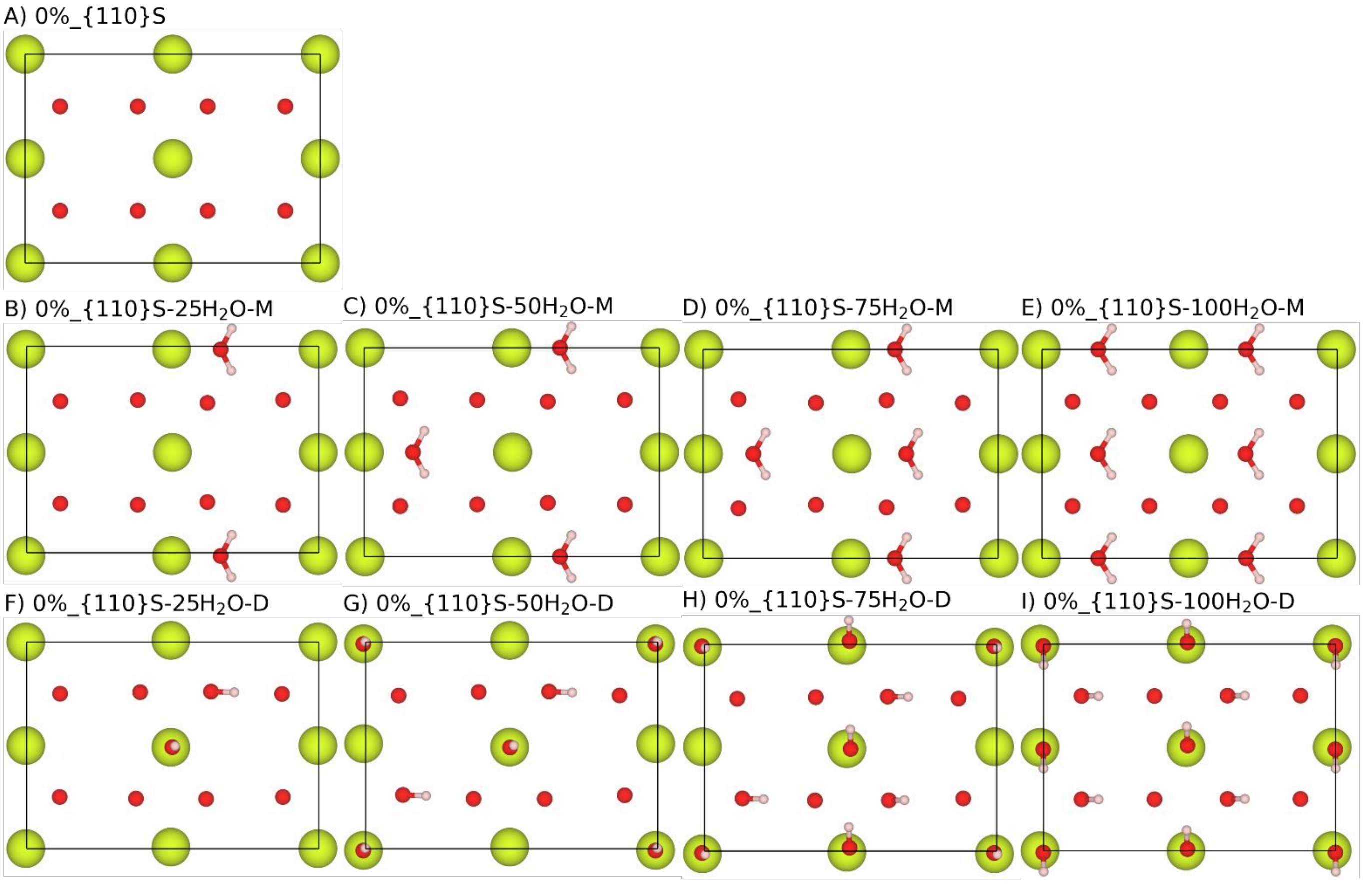

Figure S2. (A-I) Top view of the {110} CeO_2 configurations with adsorbed water.

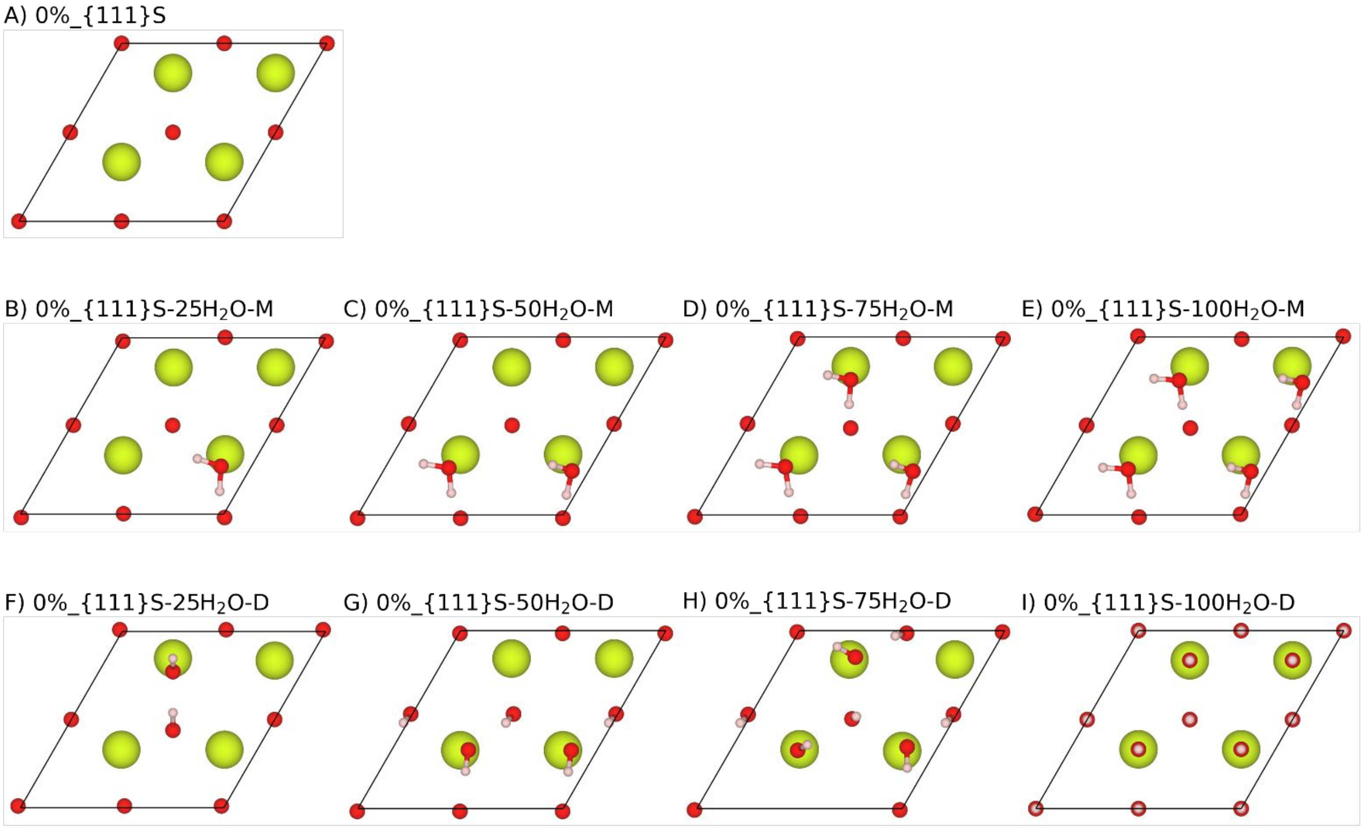

Figure S3. (A-I) Top view of the {111} CeO_2 configurations with adsorbed water.

Section S2. Hydrogen bond network on ceria surfaces

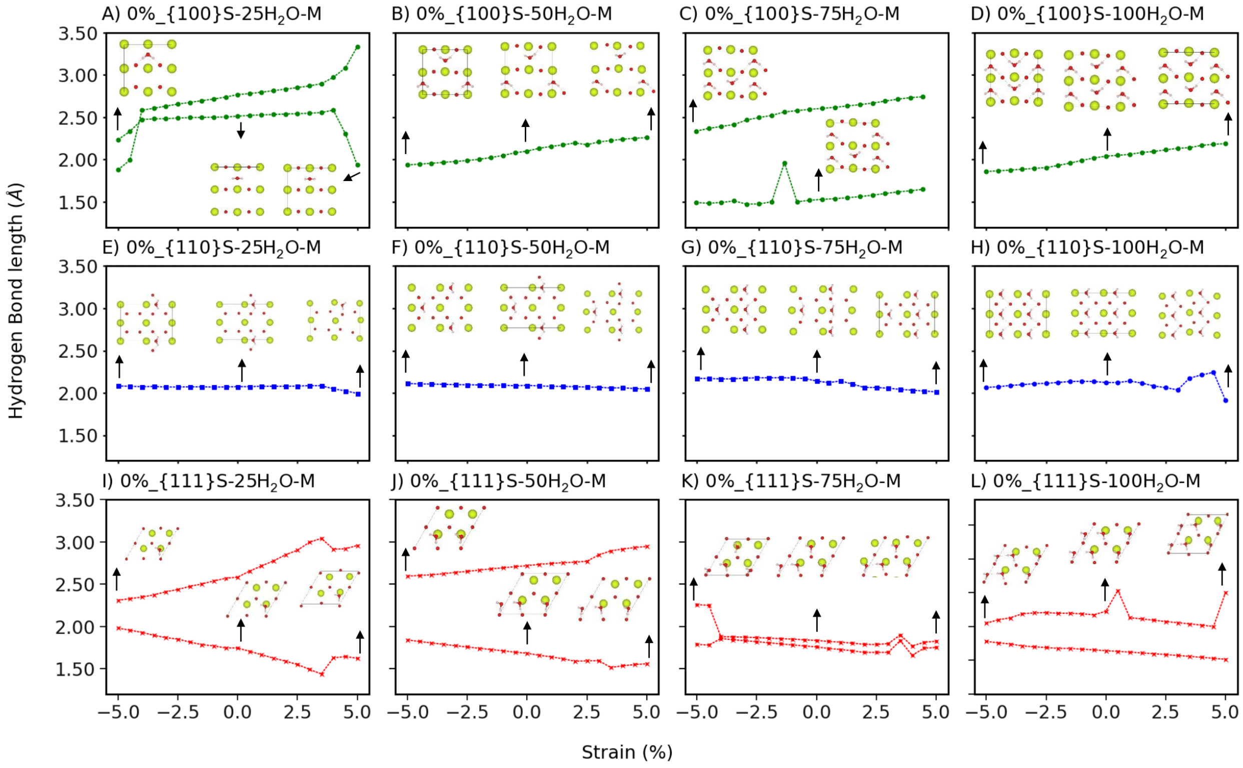

Figure S4. Average hydrogen bond length of molecular water adsorbed on ceria surfaces.

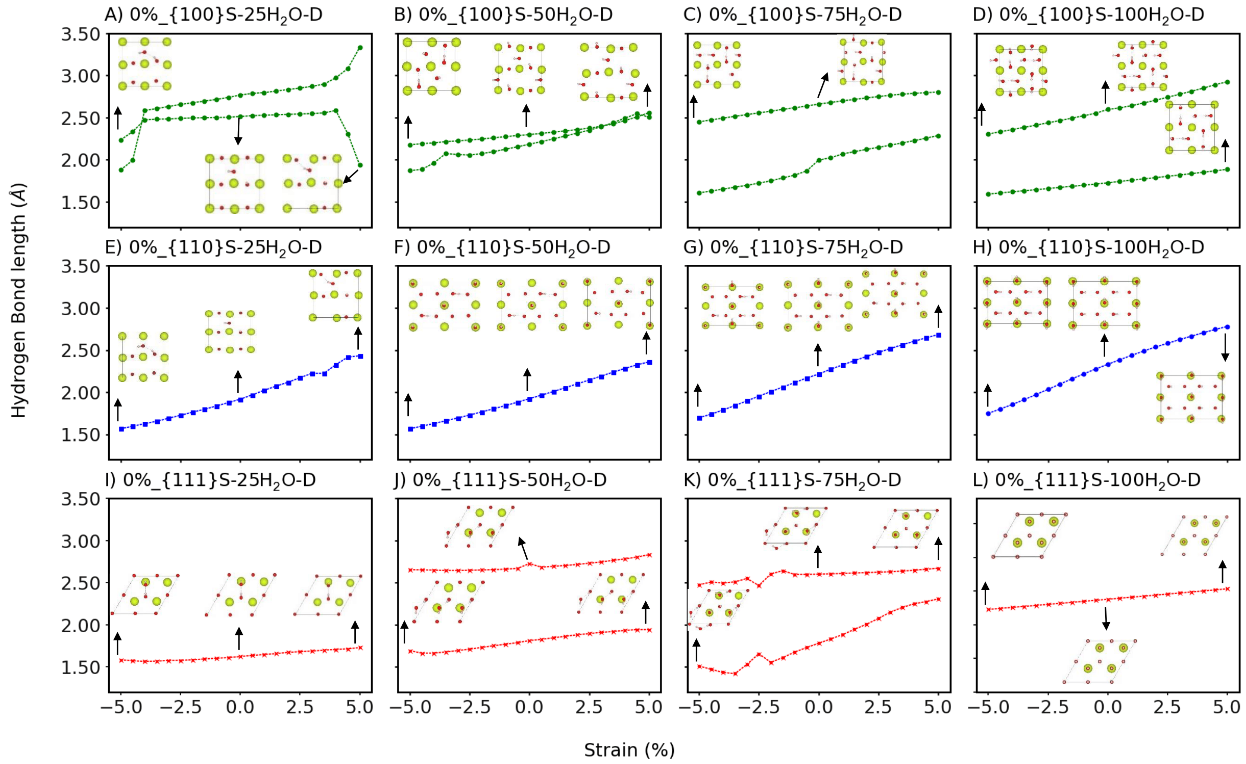

Figure S5. Average hydrogen bond length of dissociative water adsorbed on ceria surfaces.

Section S3. Desorption Temperature of water adsorbed on {100}, {110} and {111} ceria surfaces.

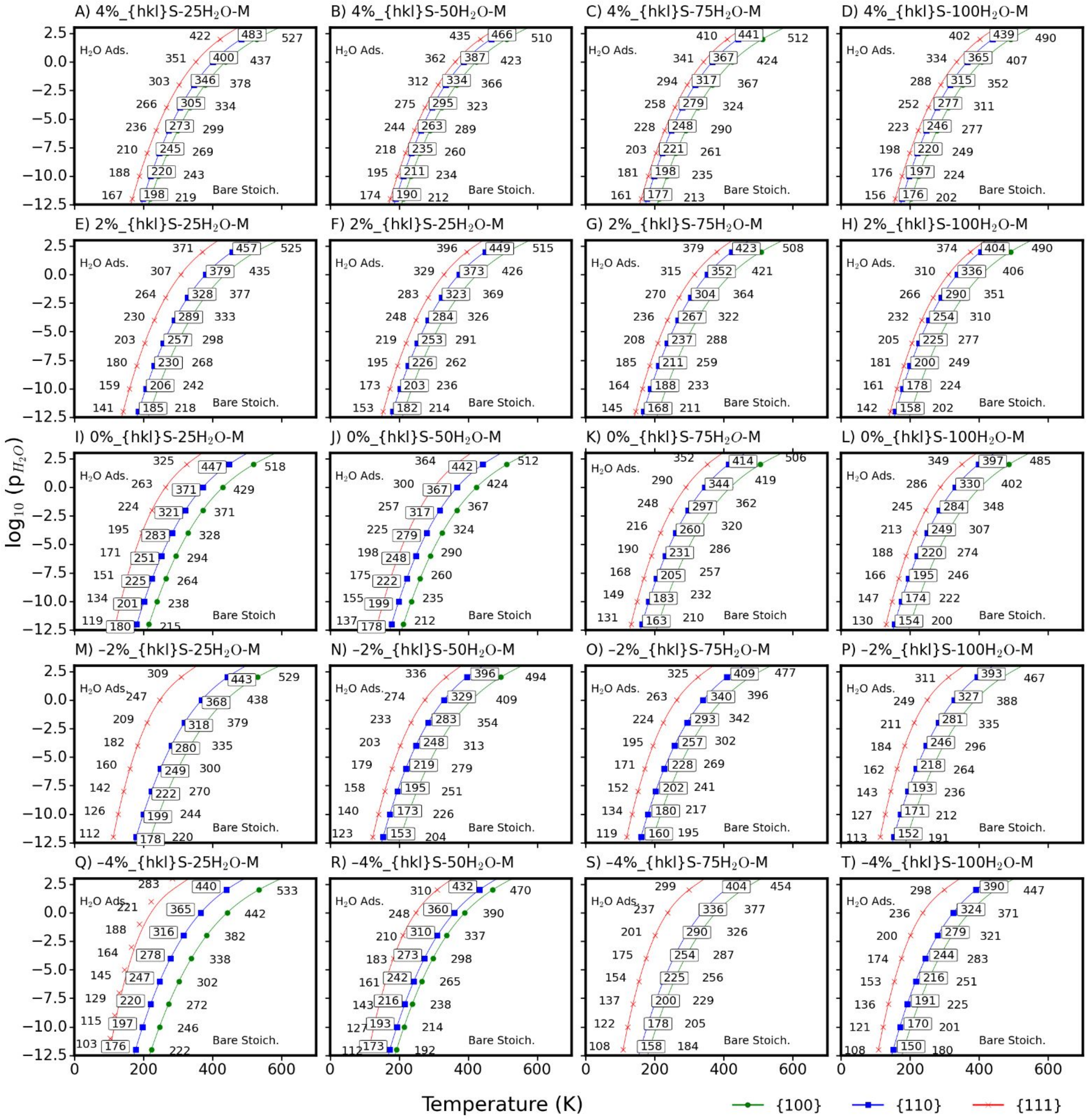

Figure S6. Predicted desorption temperatures of molecular adsorbed water on ceria surfaces.

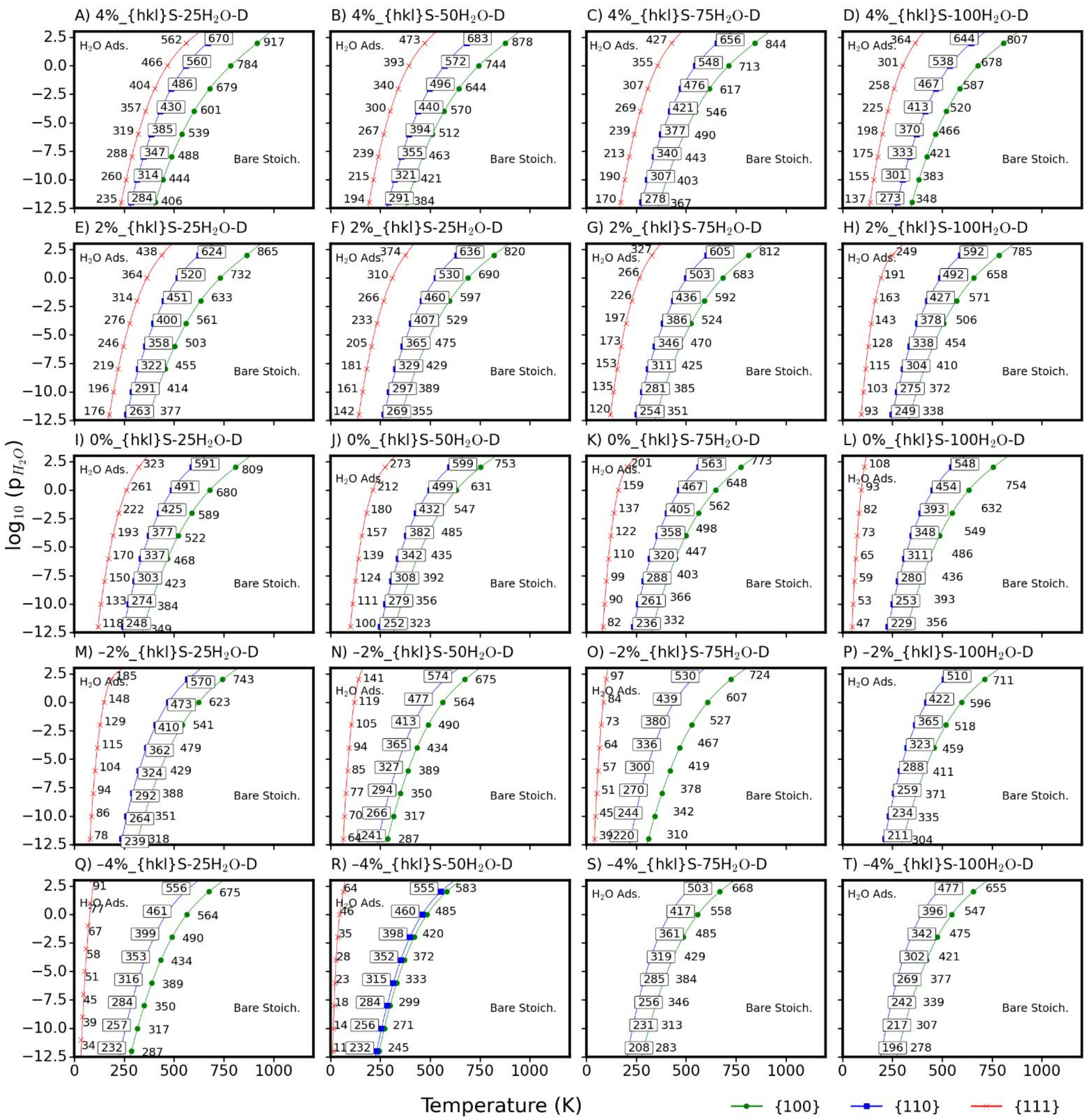

Figure S7. Predicted desorption temperatures of molecular adsorbed water on ceria surfaces.

Section S4. Surface Area phase diagrams of adsorbed water on the {100}, {110} and {111} surfaces of ceria

The “surface area” phase diagrams are presented as a function of temperature and partial pressure of water for molecularly (Figure S8-10) and dissociatively (Figure S11-S13) adsorbed water. In these diagrams the ratios between the {100}, {110} and {111} surface areas are plotted as a contour plot. Each colour represents a different ratio amongst the three surfaces and is presented alongside the colour bar close to the phase diagram. For example, a ratio {100} : {110} : {111} = 1 : 2 : 97 depicts a situation where a nanoparticle expresses 1% of the {100}, 2% of the {110} and 97% of the {111} surfaces. The three values within each ratio add up to 100%. The white dotted lines represent the predicted desorption temperatures of molecular water (Figure S8-S10) and dissociative water (Figure S11-S13). This increases the complexity but allows for the identification of the surface speciation. Any surface at lower temperatures of the temperatures of desorption is adsorbed with water, but any surface at higher temperatures of the temperatures of desorption is bare (no water is adsorbed on the surface). For example, if the ratio {100} : {110} : {111} = 1 : 2 : 97 is at conditions of temperature and pressure below the temperature of desorption of water for the {100} surface and above the temperatures of desorption for the {110} and {111} surfaces, it means that the 1% surface area of the nanoparticle that expresses the {100} surface is hydrated, while the 2% and the 97% surface areas of the nanoparticle expressing the {110} and the {111} surfaces respectively are both bare (not hydrated). Whereas the ratio of the surface areas for the {100} : {110} : {111} ratios (Figure S8-S13) shows what the percentage of each surface contributing to the shape of the nanoparticle at specific conditions of temperature and pressure, it cannot give the shape of the nanoparticle. Thus, one needs to generate the nanoparticle morphology using the Wulff construction at each condition of temperature and pressure. Nanoparticle morphologies are created for only relevant conditions of temperature and pressure in Figures S14-S17. For each nanoparticle we also provide the corresponding surface area ratio {100} : {110} : {111} and the corresponding surface composition. “H” stands for hydrated while “B” stands for bare. For example, we use our ratio {100} : {110} : {111} = 1 : 2 : 97, mentioned previously. As only the {100} surface was hydrated, while the {110} and the {111} surfaces were bare, then one would read H : B : B. If a surface is not expressed in the morphology the “-“ is present.

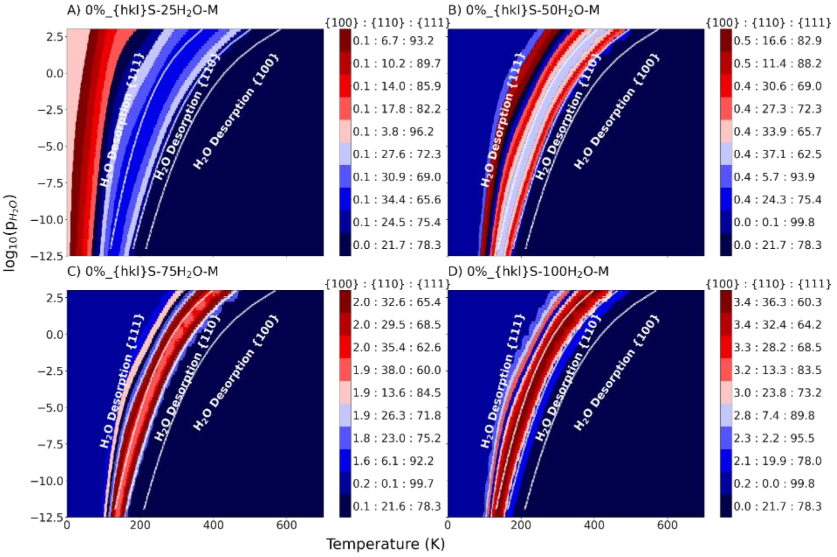

Figure S8. Predicted ratios between the {100}, {110} and {111} surface areas, when molecular water is adsorbed on the unstrained surfaces, as a function of temperature and partial pressure of water. The white dotted lines represent the predicted desorption temperatures of molecular water from the surfaces to indicate the surface speciation.

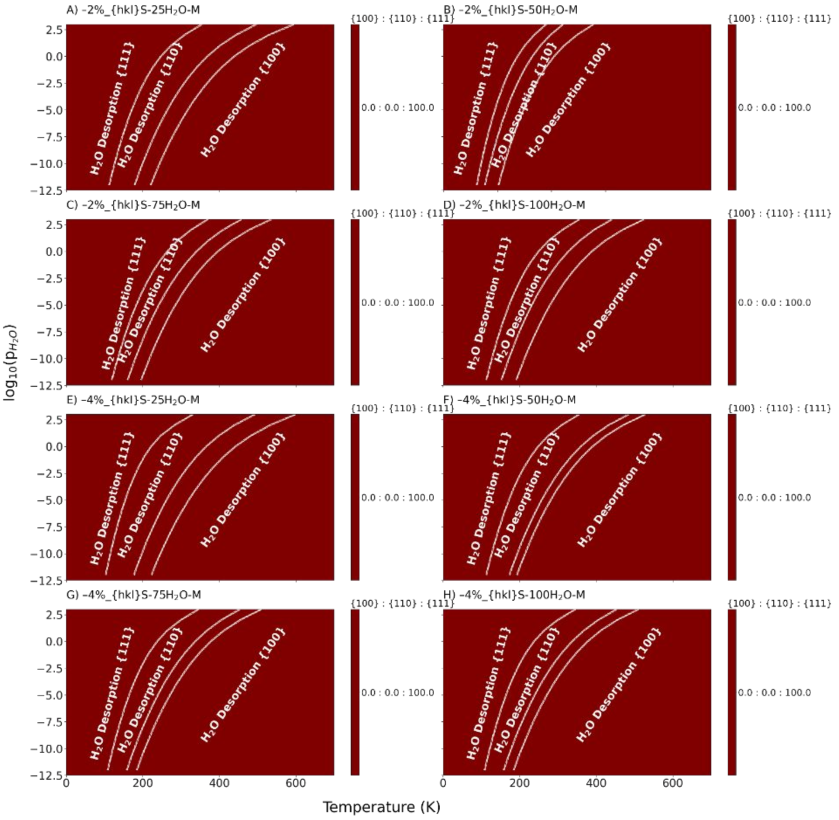

Figure S9. Predicted ratios between the {100}, {110} and {111} surface areas, when molecular water is adsorbed on the compressive strained surfaces, as a function of temperature and partial pressure of water. The white dotted lines represent the predicted desorption temperatures of molecular water from the surfaces to indicate the surface speciation.

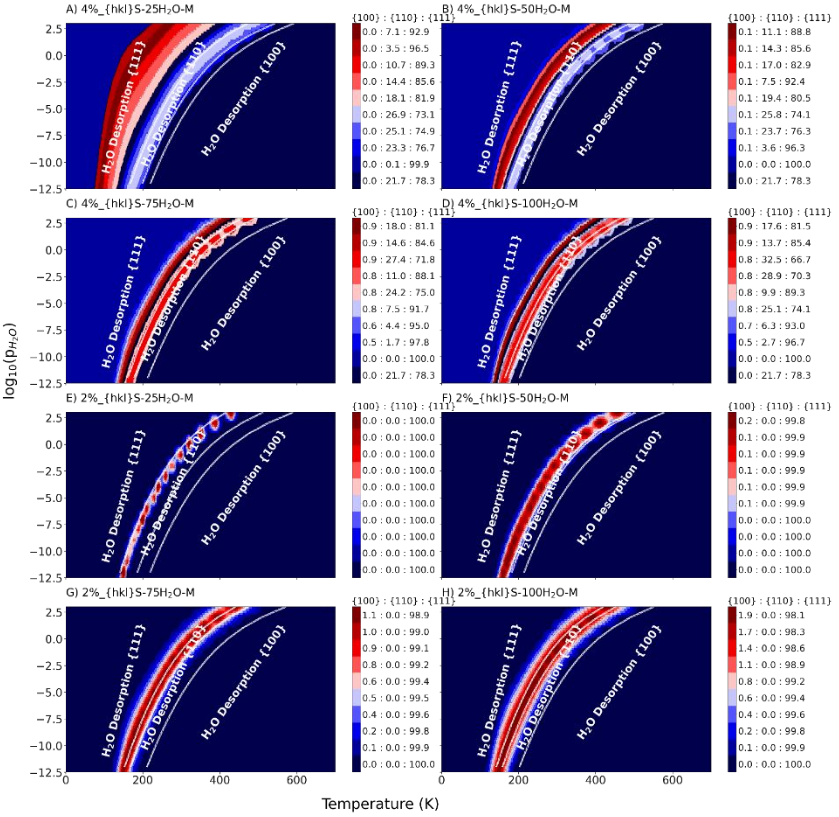

Figure S10. Predicted ratios between the {100}, {110} and {111} surface areas, when molecular water is adsorbed on the tensile strained surfaces, as a function of temperature and partial pressure of water. The white dotted lines represent the predicted desorption temperatures of molecular water from the surfaces to indicate the surface speciation.

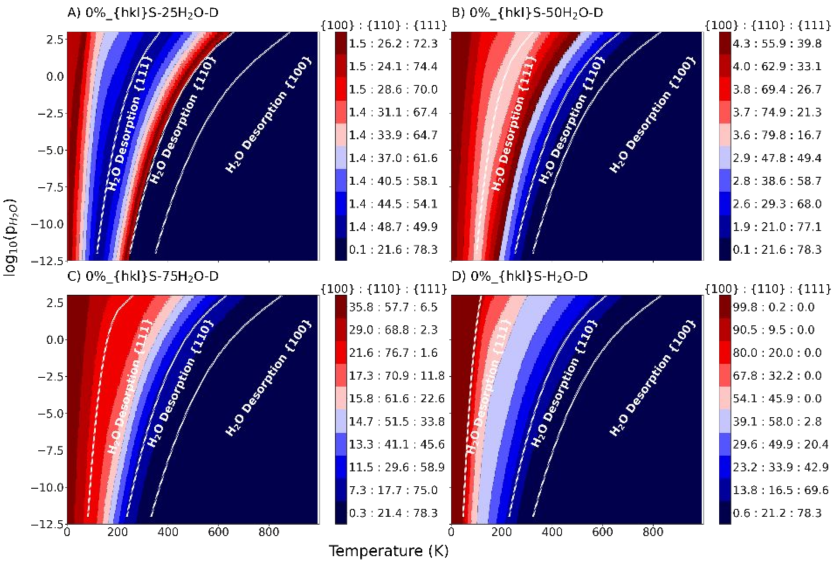

Figure S11. Predicted ratios between the {100}, {110} and {111} surface areas, when dissociative water is adsorbed on the unstrained surfaces, as a function of temperature and partial pressure of water. The white dotted lines represent the predicted desorption temperatures of dissociative water from the surfaces to indicate the surface speciation.

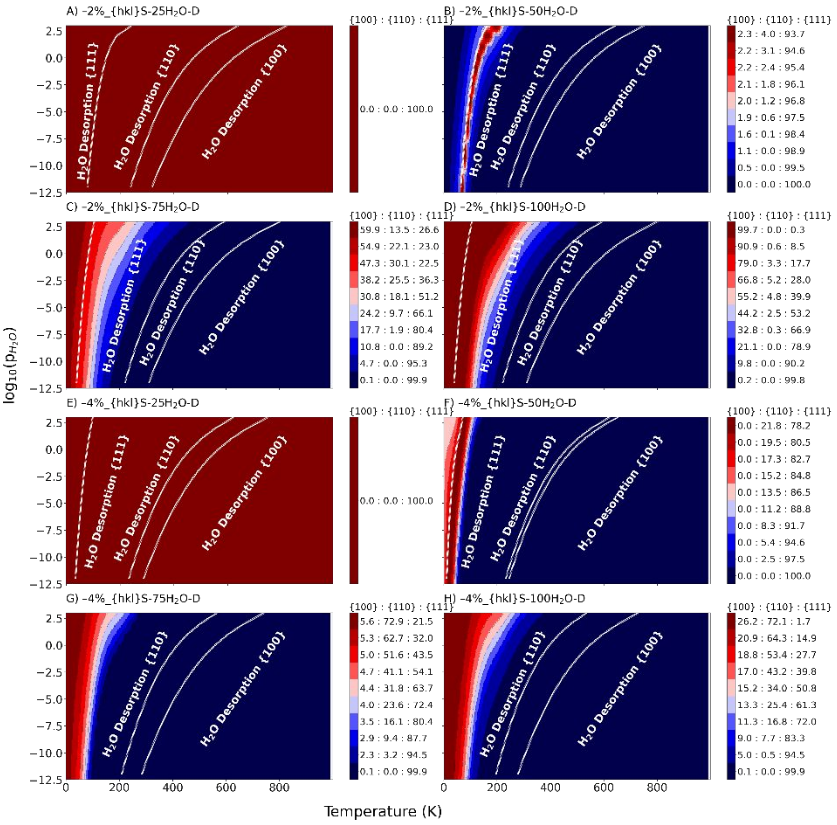

Figure S12. Predicted ratios between the {100}, {110} and {111} surface areas, when dissociative water is adsorbed on the compressive strained surfaces, as a function of temperature and partial pressure of water. The white dotted lines represent the predicted desorption temperatures of dissociative water from the surfaces to indicate the surface speciation.

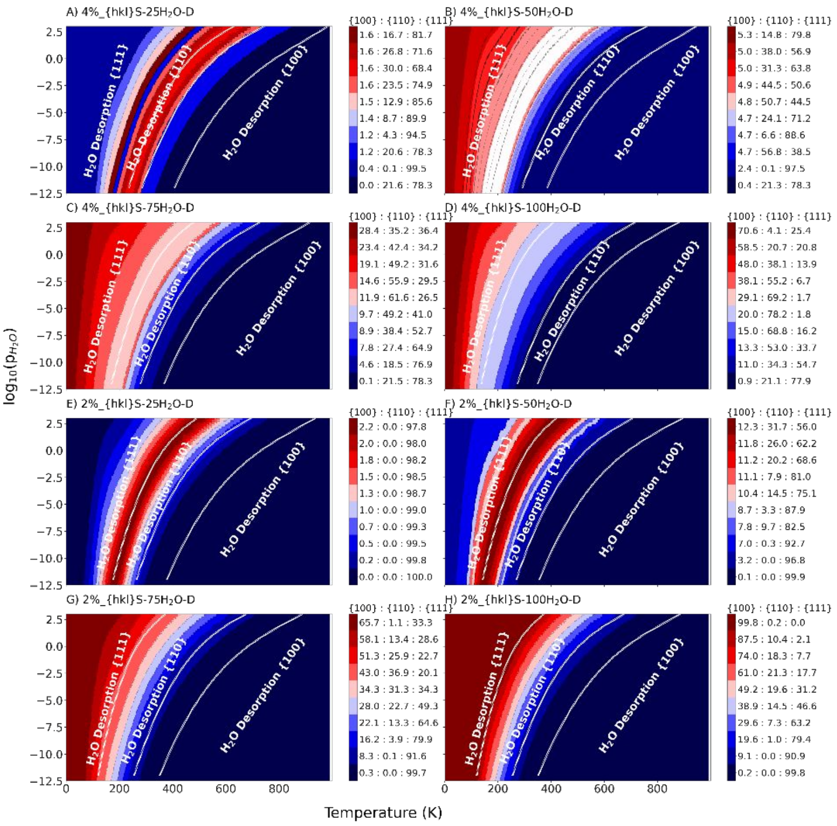

Figure S13. Predicted ratios between the {100}, {110} and {111} surface areas, when dissociative water is adsorbed on the tensile strained surfaces, as a function of temperature and partial pressure of water. The white dotted lines represent the predicted desorption temperatures of dissociative water from the surfaces to indicate the surface speciation.

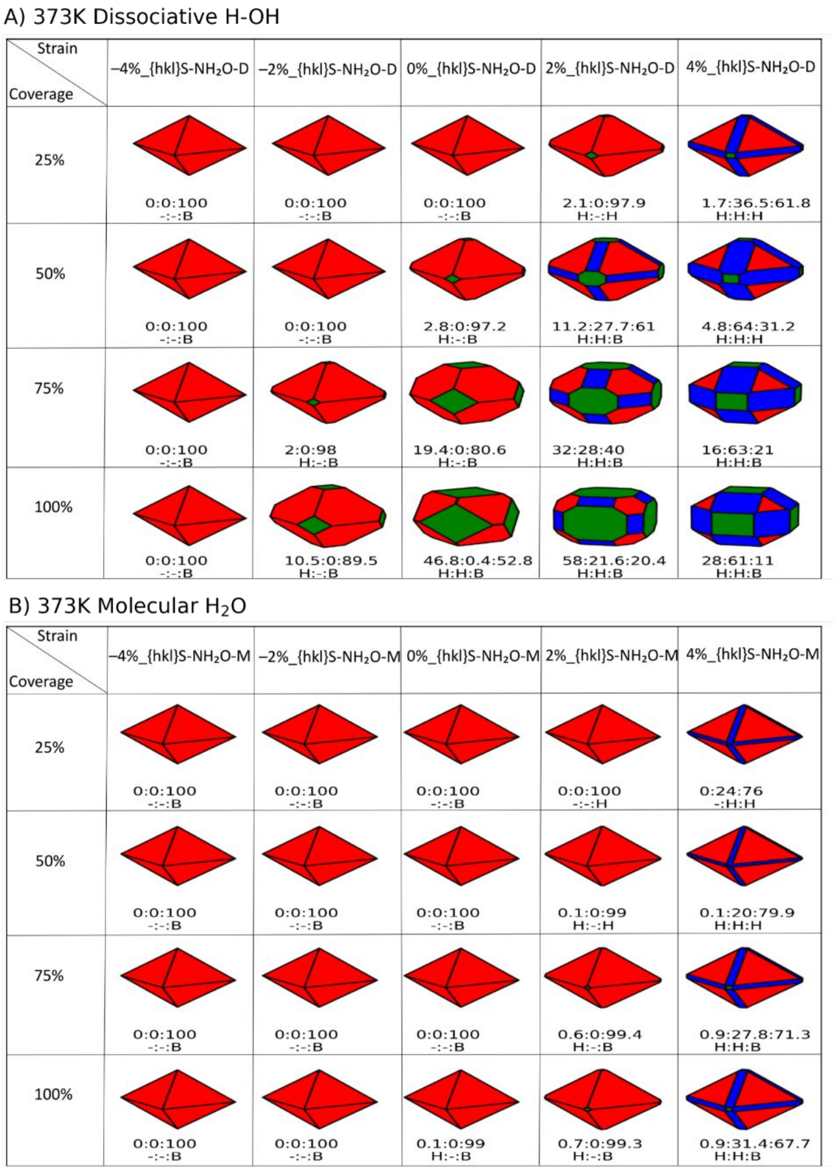

Figure S14. The shapes of CeO_2 nanoparticles in the presence of dissociative (A) and molecular (B) water at 373K. Red, blue and green represent the {111}, {110} and {100} CeO_2 surfaces, respectively. The shapes of the nanoparticles are generated at a water partial pressure of 1bar.

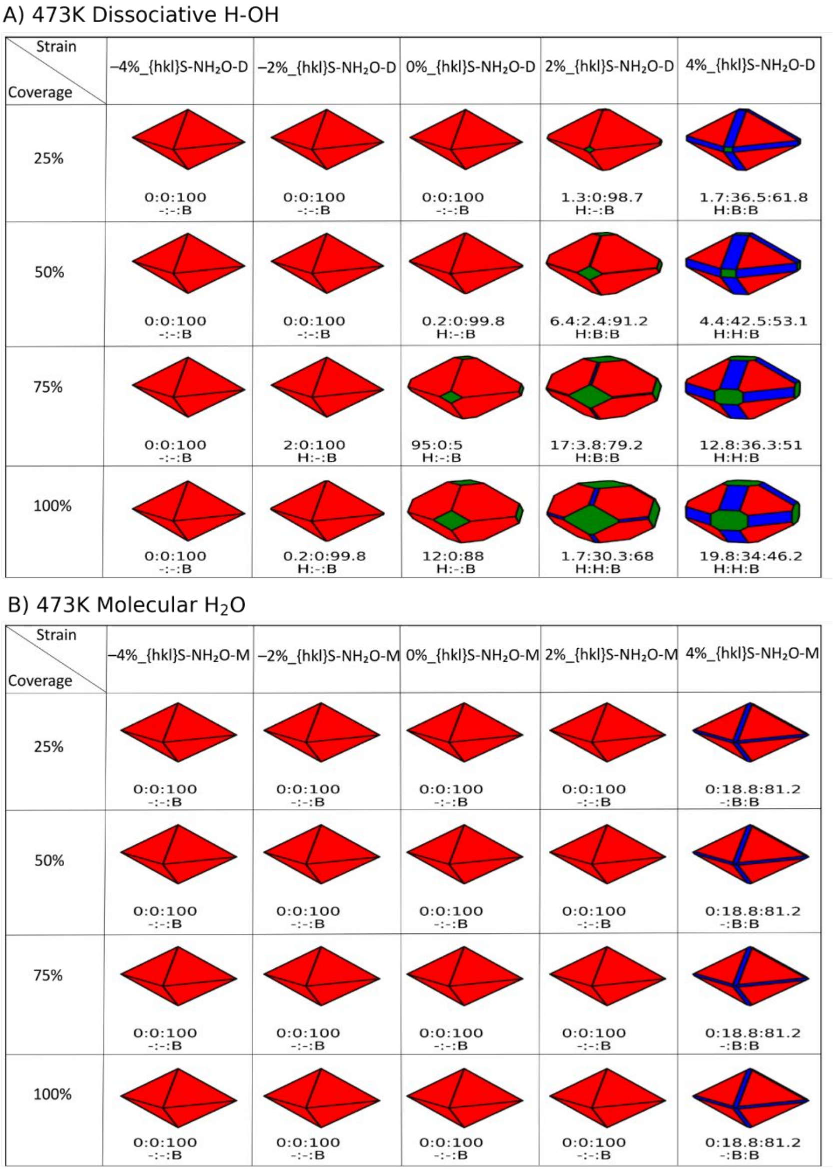

Figure S15. The shapes of CeO_2 nanoparticles in the presence of dissociative (A) and molecular (B) water at 473K. Red, blue and green represent the {111}, {110} and {100} CeO_2 surfaces, respectively. The shapes of the nanoparticles are generated at a water partial pressure of 1bar.

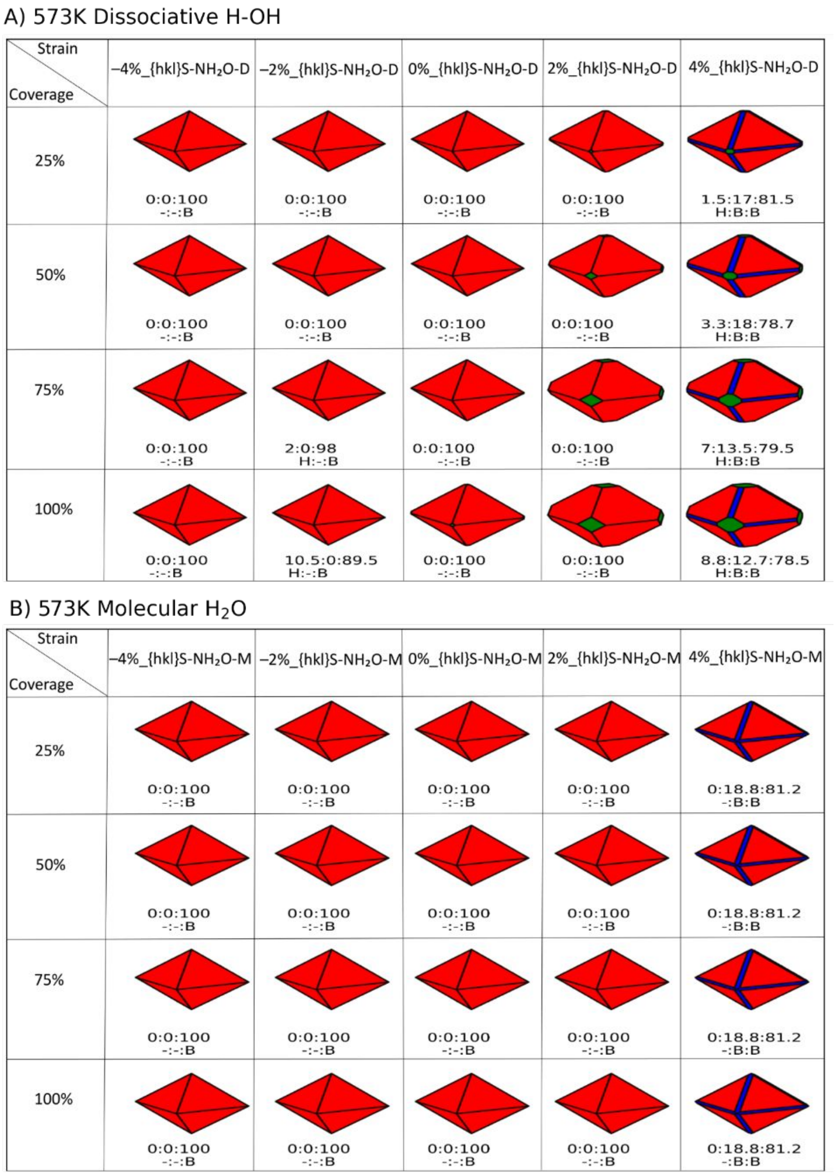

Figure S16. The shapes of CeO_2 nanoparticles in the presence of dissociative (A) and molecular (B) water at 573K. Red, blue and green represent the {111}, {110} and {100} CeO_2 surfaces, respectively. The shapes of the nanoparticles are generated at a water partial pressure of 1bar.

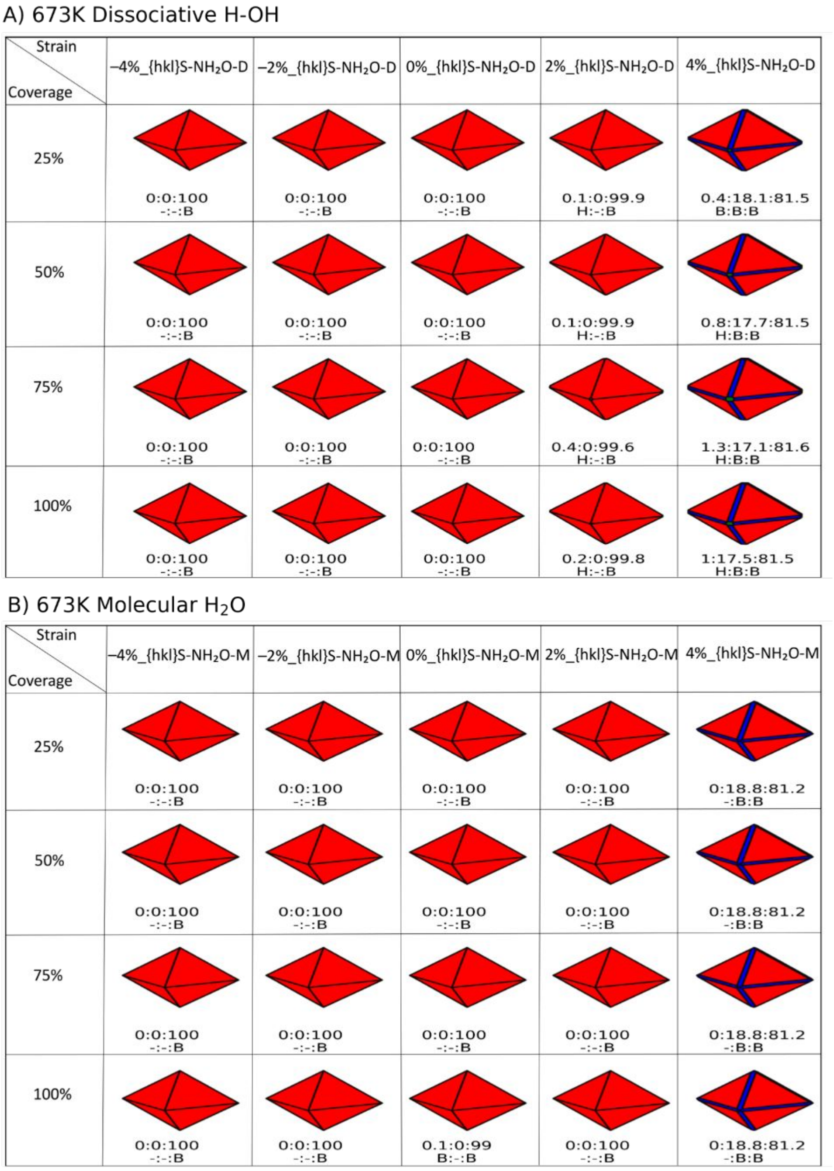

Figure S17. The shapes of CeO_2 nanoparticles in the presence of dissociative (A) and molecular (B) water at 673K. Red, blue and green represent the {111}, {110} and {100} CeO_2 surfaces, respectively. The shapes of the nanoparticles are generated at a water partial pressure of 1bar.
